# Lectin-mediated, time-efficient, and high-yield sorting of different morphologically intact nephron segments

**DOI:** 10.1007/s00424-023-02894-w

**Published:** 2023-12-13

**Authors:** Jessica Roskosch, Uyen Huynh-Do, Stefan Rudloff

**Affiliations:** Division of Nephrology and Hypertension, University of Bern and University Hospital Bern, Freiburgstrasse 15, CH-3010 Bern, Switzerland

**Keywords:** Microdissection, Kidney, Lectins, Flow sorting

## Abstract

The kidney is a highly complex organ equipped with a multitude of miniscule filter-tubule units called nephrons. Each nephron can be subdivided into multiple segments, each with its own morphology and physiological function. To date, conventional manual approaches to isolate specific nephron segments are very laborious, time-consuming, often limited to only a specific segment, and typically have low yield. Here, we describe a novel, unconventional method that is superior in many aspects to previous protocols by combining low-cost fluorophore-conjugated lectins or agglutinins (Flaggs) with flow sorting. This allows the simultaneous separation of different nephron segments with preserved 3D morphology from mouse or human samples in under 3 h. Using a 200-µm nozzle and 5 psi, glomeruli, proximal, or distal convoluted tubules are sorted with Cy3-labeled *Sambucus Nigra* agglutinin (SNA-Cy3), Fluorescein-labeled *Lotus Tetragonolobus* lectin (LTL-FITC), or Pacific Blue-labeled soybean agglutinin (SBA-PB), respectively. Connecting tubules and collecting ducts are sorted by double-positive SBA-PB and SNA-Cy3 signals, while thick ascending limb segments are characterized by the absence of any Flaggs labeling. From two mouse kidneys, this yields 37–521 ng protein/s or 0.71–16.71 ng RNA/s, depending on the specific nephron segment. The purity of sorted segments, as assessed by mRNA expression level profiling of 15 genes, is very high with a 96.1-fold median enrichment across all genes and sorted segments. In summary, our method represents a simple, straightforward, cost-effective, and widely applicable tool yielding high amounts of pure and morphologically largely intact renal tubule materials with the potential to propel nephron segment-specific research.

**Supplementary Information:**

The online version contains supplementary material available at 10.1007/s00424-023-02894-w.

## Introduction

The nephron is the central functional filter-tubule unit of the kidney. Per kidney, up to one million of these anatomically and physiologically complex units are tightly packed together in a complex network of blood vessels and interstitial cells. Together, they carry out a multitude of vital functions such as the excretion of toxins, the regulation of water, electrolyte and pH balance, and blood pressure, or the production of hormones. At its proximal end in the renal cortex, each nephron starts with a glomerulus, where blood filtration takes place, followed by a long tubule system that loops in and out of the renal medulla. A key role for the glomerular filter play particular epithelial cells called podocytes that form the slit diaphragm, a specialized intercellular junction, between miniscule foot processes of neighboring cells [13]. This highly specialized function is also mirrored by their unique gene expression profile including, among others, the filter proteins nephrin (NPHS1) and podocin (NPHS2), or the transcription factor WT1. Along its course, the tubule system of the nephron can be divided into five major segments according to morphology, physiological function, and developmental origin. In proximal–distal order, these are the proximal tubule (PT), the loop of Henle (LOH), the distal convoluted tubule (DCT), the connecting tubule (CNT), and the collecting duct (CD) [2]. Based on appearance, the PT can be further subdivided into a convoluted part (PCT) followed by a straight part (PST) which descends into the outer medulla. Characteristic for the PT is its surface-enlarging brush border membrane, where most water and urinary metabolites are reabsorbed [7]. To this end, it is equipped with a variety of pumps, transporters, and receptors. Typical examples of these transport proteins only found in the PT are the sodium-dependent phosphate transport proteins 2A or 2C (encoded by *SLC34A1* or *SLC34A3*, respectively), or the receptor megalin aka low-density lipoprotein receptor-related protein 2 (LRP2) [2]. The main function of the LOH is to enhance the urine concentration capability of the kidney by increasing the tonicity of the medullary interstitium [2]. In its descending (thin) limb, water diffuses into the hyperosmolar medullary interstitium, which in turn is generated by the active transport of sodium into this interstitial space by the cells of the water-impermeable thick ascending limb (TAL). The TAL-specific, furosemide-sensitive Na–K-Cl cotransporter NKCC2 (encoded by *SLC12A1*) plays an important role for the reabsorption of these ions from the tubular fluid [4]. Uromodulin (UMOD), the most abundant protein in human urine, is synthesized and secreted by TAL cells, and to a lesser degree also by the proximal portion of the DCT, and plays an important role in the defense against pathogens in urine. On the other hand, *UMOD* missense mutations cause autosomal dominant tubulointerstitial kidney disease (ADTKD), one of the most common monogenic kidney diseases [29]. At its distal end, a group of tightly packed cells (the macula densa) forms the connection of the TAL with the DCT [20], the shortest segment of the nephron. The DCT, which can be subdivided into a proximal DCT1 and a distal DCT2, plays an important role in the homeostasis of potassium, sodium, magnesium, and calcium [30, 34]. A key marker of the entire DCT is the thiazide-sensitive Na-Cl cotransporter NCC (encoded by *SLC12A3*). DCT1 is further characterized by the expression of intracellular calcium-binding protein parvalbumin (PVALB) and the magnesium channel TRPM6. On the other hand, DCT2 is marked by the intracellular calcium-binding protein calbindin (CALB1) and the basolateral Na-Ca exchanger NCX1 (encoded by *SLC8A1*). CALB1 and NCX1 are also found in the CNT that connects the DCT2 to the CD. In addition to these calcium transport proteins, expression of subunits of the amiloride-sensitive sodium channel ENaC (e.g., SCNN1G) begins in this segment and continues in the CD. Recent studies using single-cell sequencing profiles [2, 6] have begun to challenge the classical separation of DCT2 and CNT as two distinct entities and propose to define this nephron segment based on its unique marker expression of calcium transporters in addition to the overlapping expression of DCT and CD markers (NCC and SCNN1G, respectively). The CD is comprised of two distinct cell types called principal or intercalated cells [25, 28]. The main task of the principal cells is the finetuning of sodium and water reabsorption mediated by ENaC and the water channel aquaporin 2 (AQP2), respectively. Intercalated cells of type A aid to acidify the urine by excretion of protons, while type B intercalated cells expressing the Cl-HCO3 exchanger pendrin (encoded by SLC26A4) contribute to raise the urinary pH. In order to properly fulfill the broad range of functions described above, each nephron segment is interdependent on the correct function of the other segments. A disruption of these interwoven processes in any nephron segment will ultimately affect the entire nephron and if left untreated can lead to severe illnesses and in the long term to permanent renal functional decline, summarized as chronic kidney disease (CKD). On the other hand, a given treatment will also affect each nephron segment in a different way. Thus, since such segment-specific (patho)mechanisms remain poorly understood, the elucidation of segment-specific processes and how they contribute to renal disease initiation, progression, or resolution is an unmet medical need. To address this, novel, cost-effective research tools must be developed that provide large quantities of ready-to-use segment-specific renal tubule material for unbiased conduction of transcriptomic, epigenomic, proteomic, or metabolomic analyses. Given this, it is all the more astonishing that even the most sophisticated studies still rely on manual microdissection [15, 16, 29] or immunodissection [12, 35] of nephron segments using protocols that were partly established more than 30 years ago [45]. Besides high costs, these protocols are not only very time-consuming and laborious but often limited to one segment or individual cells [12]. In this study, we now describe a novel technique to simultaneously isolate different, morphologically largely intact nephron segments from the same kidney using fluorescently labeled lectins or agglutinins (Flaggs) and flow sorting. Lectins have first been described in the nineteenth century by P. Stillmark [41], who reported on the ability of ricin, a protein isolated from castor beans, to agglutinate red blood cells. Following this initial discovery, several similar proteins were identified mostly in grains and beans [32, 41]. In this study, we assessed Jacalin (jackfruit), SNA (*Sambucus Nigra*—black elderberry), PNA (peanut), DBA (*Dolichos Biflorus*—horse gram), SBA (soybean), and LTL (*Lotus Tetragonolobus*—winged pea). In 1952, the agglutination activity of these proteins was ascribed to their carbohydrate-binding activity [44]. At around the same time, the term “lectin” derived from Latin *legere* for select or choose was introduced [3]. Since then, lectins have been intensively used for identifying cell surface sugar moieties under physiological and pathophysiological conditions [32]. The kidneys of humans [8, 9, 39], mice [31], and rats [21, 31] have been widely mapped in regard to their glycosylation state. Such glycome maps helped to identify pathological differences in polycystic kidney disease [17], the differential diagnosis of renal tumors in children [14], or during renal aging [11]. Surprisingly, despite the knowledge of altered glycosylation states in different pathological settings [11, 14, 17], only a few publications have investigated the molecular mechanisms or functional consequences that accompany this alteration in renal diseases [1, 33, 38]. Taken all together, we subject Flaggs-labeled nephron segments to flow sorting with the aim to provide an alternative for the established manual microdissection protocols of nephron segments [10, 37, 45]. On the one hand, our novel protocol reduces time, labor, and costs, and on the other hand provides from the same kidney large yields of pure, morphologically largely intact nephron segments with PT, TAL, DCT, and CNT/CD identity as well as glomeruli.

## Methods

### Animals

All animal experiments were conducted according to the Swiss law for the welfare of animals and were approved by the local authorities (Canton of Bern BE3/11 and BE106/20). C57BL/6N mice were housed at 23 °C and 50–60% humidity in IVC cages with free access to chow and water and a 12-h day/night cycle. We used four female and five male mice, aged 13–47 weeks for our study.

### Human samples

Samples of fresh human kidney tissue (taken from the apparently healthy part of nephrectomized kidneys from four patients with renal cell carcinoma) were provided by the Clinic for Urology at the University Hospital Bern, Switzerland. Before nephrectomy, in accordance with the Swiss Human Research Act, these patients gave their general consent for the further use of tissue samples for research projects including their consent to publish the results obtained with these samples.

### Covalent labeling of SBA with Pacific Blue

Soybean agglutinin (SBA; L-1010, Vector Laboratories) was conjugated to the fluorophore Pacific Blue™ (PB) using the Protein Labeling Kit from Invitrogen (P30012) according to the manufacturer. Briefly, 500 µl of the unconjugated SBA solution (2 mg/ml) was mixed with 50 µl 1 M sodium bicarbonate solution and transferred to a vial containing the reactive dye. After 60-min incubation in the dark, SBA-PB was purified using the provided spin column. The degree of labeling was determined by measuring the absorbance of PB-SBA at 280 nm and 410 nm (specific for PB).

### Fluorescent staining

Air-dried kidney sections from unfixed, fresh-frozen tissue were transferred into PBS, followed by blocking in 2% BSA in PBS for 30 min and three 2-min washing steps in PBS for 2 min. Then, the sections were either incubated only with fluorophore-conjugated lectins (2 µg/ml) or in combination with unconjugated or fluorophore-conjugated primary antibodies (1:20,000 rabbit anti-AQP2, 1:2000 rabbit anti-NCC, or 1:20,000 rabbit anti-NKCC2, kindly provided by Jan Loffing, Zurich [43]; 1:100 mouse anti-AQP2-Alexa488, sc515770 AF588, SCBT; 1:100 mouse anti-LRP2-Alexa546, sc-515772 AF546, SCBT) in PBS for 1 h at RT. After another round of washing, sections with unconjugated primary antibodies were incubated with fluorophore-coupled secondary anti-rabbit antibodies (Cy3 1:250, 711–165-152, or Cy2 1:100, 711–225-152, both Jackson ImmunoResearch) in PBS for another 30 min at RT, followed by three washes in PBS. Finally, the sections were mounted in MOWIOL solution (2.4 g MOWIOL 4–88 reagent (475904; Merck) in 6 g glycerol and 18 ml 0.13 M Tris pH 8.5) for imaging. An overview of lectins used in this study is given in Supplementary Table 1.

### Isolation and labeling of tubular nephron segments

Both kidneys were isolated immediately after euthanasia of the animals and transferred to a Petri dish containing 10 ml ice-cold dissection solution (1 × HBSS (14025–050, Gibco), 15 mM HEPES, 10 mM D-glucose, 5 mM glycine, 1 mM L-alanine, 8.75 mM D-mannitol) [10, 37]. After removing the residual fat and kidney capsule, the entire kidneys were randomly cut into small pieces of 1–2 mm^3^ using scissors. The pieces were then transferred into a 50-ml conical tube, and the remaining dissection solution was carefully removed and replaced by 10 ml of freshly prepared, sterile-filtered digestion solution (1 mg soybean trypsin inhibitor (93620, Sigma) and 10 mg collagenase type IA (C9891, Sigma) in dissection solution) [10, 37]. The tightly closed tube was placed into a shaking water bath at 37 °C for 15 min. During this time, the dissociation was enhanced by carefully pipetting up and down the kidney fragments 10 times every 5 min. The tubular nephron segments were then subsequently filtered into clean 50-ml tubes first through a 250-µm pore size membrane (PA-5XXX-250, SEFAR NYTAL®) and secondly through a 100-µm cell strainer (352360, VWR), followed by centrifugation at 350 rpm (20 g) for 2 min. The turbid supernatant containing single cells and smaller nephron fragments was carefully removed not to disturb the pelleted tubule segments. The tubular segments were then twice washed with 5 ml ice-cold BSA solution (100 mg BSA (9418, Sigma) and 1 mg soybean trypsin inhibitor (93620, Sigma) in 10 ml dissection solution) [10, 37] and pelleted at 20 g, followed by a final wash in 5 ml ice-cold dissection solution. After a last centrifugation step at 20 g and careful removal of the supernatant, the tubules were resuspended in 2 ml freshly prepared Flaggs solution (2.5 µl LTL-FITC (L32480, Invitrogen), 2.5 µl SNA-Cy3 (CL-1303, Vector Laboratories), and 1.25 µl SBA-PB (L1010, Vector Laboratories; in-house labeled with Pacific Blue) in dissection solution) and transferred into a 2-ml microcentrifuge tube. The tube was placed into a vertical rotator and staining proceeded at 5 rpm at 4 °C for 60 min. Thereafter, the tubule segments were pelleted at 30 g for 2 min in a pre-chilled tabletop microcentrifuge, once washed with dissection solution, and stored in dissection solution on ice until sorting. Fresh human kidney samples were treated like mouse tissue, with the exception that 10 ml digestion solution contained 20 mg collagenase type IA (C9891, Sigma), 20 mg collagenase type II (C2-28, Sigma), and 2 mg hyaluronidase (H3506, Sigma).

### Imaging

Fluorescence imaging was performed on an iMIC digital microscope (FEI, Type 4001) using the Polychrome V light source, an Orca-R2 camera controller from Hamamatsu (C10600), and Live Acquisition software (FEI, version 2.6.0.14). Image analysis was performed using Offline Analysis software (FEI). Single-channel images are displayed in grayscale; merged images are displayed in false colors to avoid red-green combinations.

### Tissue sorting

Sorting of nephron segments was done based on forward vs. side scatter and PB, FITC, and Cy3 with a MoFlo ASTRIOS EQ (Beckman Coulter, Indianapolis, IN, USA). Samples were passed through a 100-µm cell strainer (352360, VWR) immediately before sorting using a 200-µm nozzle and at 5-psi system pressure. Sorting precision was set to “Purify” and 1–2 droplet envelope for high purity and accurate numbers of sorted nephron segments. Sorting efficiency was about 60–70%. 1.5-ml microcentrifuge tubes containing 0.3 ml of dissection solution were used for collection. Both samples and collection tubes were kept at 6 °C throughout the sorting process.

### Post-sort viability

Trypan blue solution (T8154, Sigma) was diluted 1:2 in dissection solution and filtered through a 0.22-µm filter (SLGP033RS, Merck). A total of 20 µl of the sorted tubule segment suspension was mixed with 80 µl of the freshly prepared Trypan blue solution and incubated at RT for 10 min before visual analysis.

### RT-qPCR

Sorted tubule segments were pelleted at 1000 g for 5 min at 4 °C, and total RNA was isolated using TRI reagent (T9424, Sigma) according to the manufacturer. RNA concentration and quality were determined with a Nanodrop 1000 spectrophotometer (Thermo). RNA was transcribed into cDNA using the PrimeScript RT Reagent Kit (RR037A Takara). cDNA was diluted to 0.5–1 ng/µl (depending on the final yield), and qPCR was performed with Fast SYBR Green Master Mix (4385612, Applied Biosystems) and corresponding gene-specific primers on a QuantStudio 1 System (Applied Biosystems). Primer sequences are listed in Supplementary Table 2. The qPCR results were analyzed with Microsoft Excel using the ΔΔCt method to calculate the relative expression levels for each analyzed gene. Graphs and statistical analysis were made with GraphPad Prism 9.4.1.

### Protein yield

Sorted tubule segments were pelleted at 1000 g for 5 min at 4 °C and lysed in 100 µl ultrapure water containing 0.05% Triton X-100 (T8787, Sigma). The protein content was then determined according to Pierce Bradford Plus Protein Assay Kit (23236, Thermo). The protein yield per second was then calculated by dividing the protein amount by the number of sorted events and multiplied by the events per second.

## Results

### Binding specificities of Flaggs on fresh-frozen mouse kidney sections

**Fig. 1:**
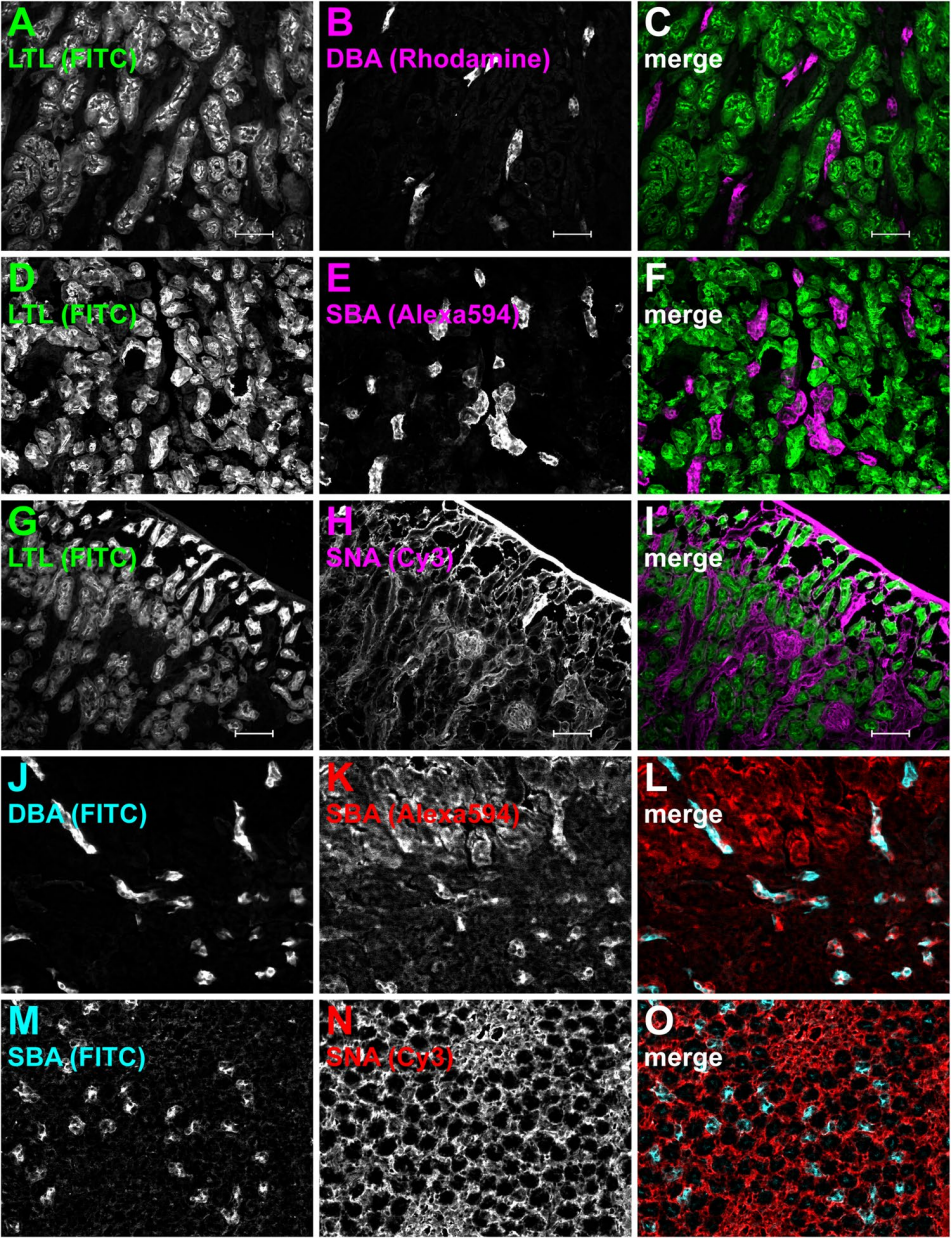
Co-staining of different Flaggs on mouse kidney sections. Cryosections were stained with different combinations of two differently conjugated Flaggs: LTL-FITC and DBA-Rhodamine (**A**–**C**), LTL-FITC and SBA-Alexa594 (**D–F**), LTL-FITC and SNA-Cy3 (**G**–**I**), DBA-FITC and SBA-Alexa594 (**J**–**L**), and SBA-FITC and SNA-Cy3 (**M**–**O**). In merged images, LTL-FITC is always shown in green, while other FITC-conjugates are always depicted in cyan. To avoid red-green signals in merged images, Rhodamine, Alexa, or Cy3 is shown in either red or false-color magenta. Single channels are shown in grayscale. LTL did not show overlapping staining with DBA, SAB, or SNA. SBA showed partial overlap with DBA or SNA. Scale bars 100 µm

As a first step, we aimed to determine the binding specificity of six different fluorophore-tagged lectins or agglutinins (Flaggs) on unfixed, fresh-frozen kidney sections. A complete list of Flaggs used in this study is given in Supplementary Table 1. Importantly, for staining, all Flaggs were diluted in PBS only, as BSA in the staining solution appeared to interfere with the staining process. This was most likely due to the extensive absorption of some Flaggs by the BSA in solution. LTL was located to the apical membrane of most tubules in the cortex (Fig. 1A, D, and G, and Supplementary Fig. 1D). DBA showed specific staining of few tubule segments in the cortex and the outer medulla (Fig. 1B and J, and Supplementary Fig. 1A). SBA stained some tubule segments in the cortex and the apical side of tubule in the inner and outer medulla (Fig. 1E, K, and M, and Supplementary Fig. 1G). In addition to glomeruli (Fig. 1H), SNA also labeled the luminal side of some tubule segments traversing the cortex and outer medulla (Fig. 1N and Supplementary Fig. 1 J). PNA showed specific luminal staining of tubule segments in the inner and outer medulla (Supplementary Fig. 1B, H, and K) and almost all tubules in the cortex (Supplementary Fig. 1E). In contrast to all other Flaggs, Jacalin staining was uniformly present (not shown), and it was therefore excluded from further use. In a next step, we performed double Flaggs staining to assess potential combinations that could be used for sorting. Non-overlapping patterns were found for LTL/DBA (Fig. 1A–C), LTL/SBA (Fig. 1D–F), and LTL/SNA (Fig. 1G–I). All other Flagg combinations showed either partial or complete overlap. For the pair LTL and PNA, only some tubules were stained by PNA alone (dominant), while LTL completely overlapped with PNA (Supplementary Fig. 1D–F). Similar patterns with one dominant Flagg (mentioned first) were found for SBA/DBA (Fig. 1J–L), PNA/DBA, or PNA/SBA (Supplementary Fig. 1A–C and G–I). SBA and SNA exhibited a co-dominant staining pattern (Fig. 1M–O), while PNA and SNA signals overlapped completely (Supplementary Fig. 1 J–L). Table 1 provides an overview of the different staining patterns of the different Flagg pairs.

**Table 1:** Flaggs combinability chart for mouse kidney

	DBA	LTL	PNA	SBA	SNA
DBA		–	+ _P_	+ _P_	nd
LTL	–		+ _P_	–	–
PNA	+	+		+	+ _P_
SBA	+	–	+ _P_		+ _P_
SNA	nd	–	+	+ _P_	

No overlap (–), complete overlap ( +), partially overlap (+ _P_), not determined (*nd*). For + _P_, non-overlapping staining was detected for the Flaggs heading the column

Since PNA showed partial or complete overlap with all other Flaggs, we decided to exclude it from further use at this stage. DBA-Rhodamine was also excluded from further use as it seemed to leak into the far-red channel. Thus, for the rest of the study, we worked with the three remaining Flaggs LTL, SBA, and SNA.

### Triple Flaggs staining of freshly isolated nephron segments

**Fig. 2:**
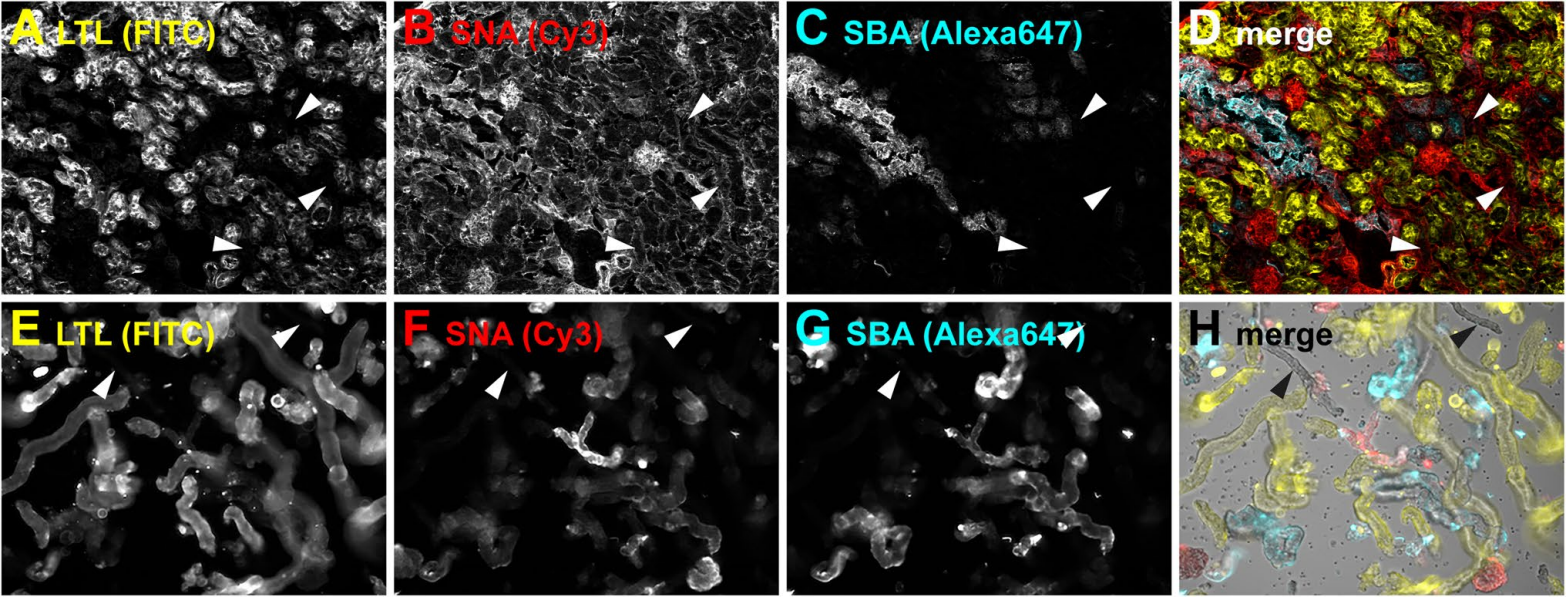
Triple Flaggs staining of freshly isolated mouse nephron segments. Cryosections (**A**–**D**) or freshly isolated tubules (**E**–**H**) were stained with LTL-FITC (**A**, **E**), SNA-Cy3 (**B**, **F**), and SBA-Alexa647 (**C**, **G**). To avoid red-green signals in merged images, FITC is shown in yellow. Cy3 in red and Alexa647 in cyan. Single channels are shown in grayscale. Besides glomeruli (red), four differently stained tubule segments can be seen: large yellow tubules, small-sized red and cyan tubules, large cyan tubules, and unstained tubules (marked by arrowheads)

Before proceeding to stain freshly isolated nephron segments, we tested the staining performance of the three Flaggs LTL-FITC, SBA-Alexa647, and SNA-Cy3 on fresh-frozen kidney sections (Fig. 2A–D). The triple stain revealed that SBA and SNA only partially filled the gaps in LTL-FITC negative areas and that some tubule segments still remained largely unstained (arrowheads in Fig. 2D). Staining of freshly isolated nephron segments with this triple combination showed very comparable results (Fig. 2E–H). Most large-diameter tubule segments were stained with LTL-FITC (Fig. 2E), and a minority with SBA-Alexa647 (Fig. 2G). Glomeruli were stained with SNA-Cy3 (Fig. 2F), and small- or medium-sized tubule segments were stained with either SBA-Alexa647 or SNA-Cy3, or both. Importantly, some nephron fragments showed no fluorescence (black arrowheads in Fig. 2H). Taken together, with the help of the Flaggs combination of LTL, SBA, and SBA, at least four different tubule segments (LTL-positive, SBA-positive, SNA/SBA-double positive, and LTL/SBA/SNA-triple negative) and glomeruli could be sorted.

### Co-staining of Flaggs and nephron segments markers

**Fig. 3:**
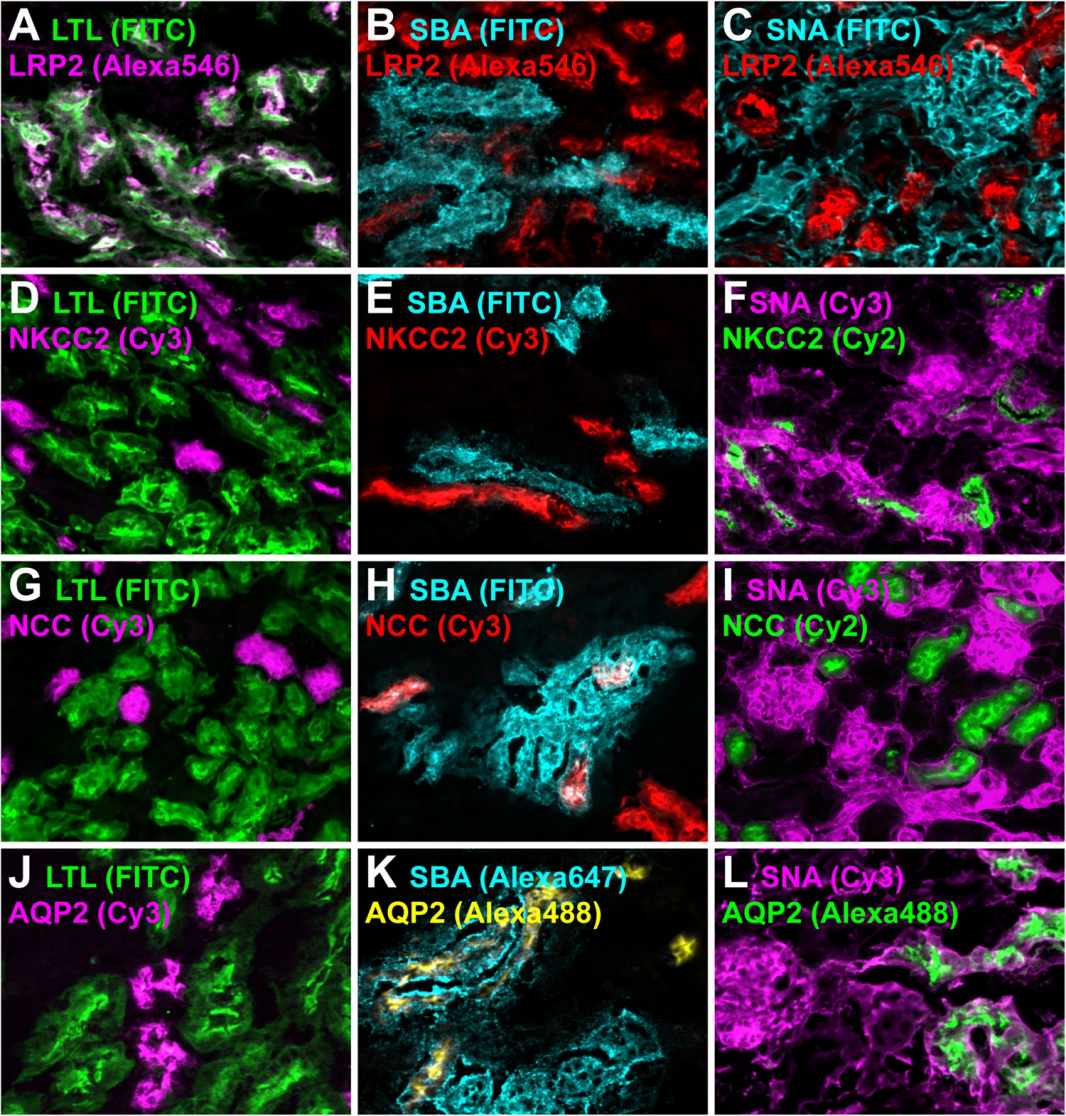
Staining specificity of Flaggs on mouse kidney sections. Cryosections were stained with LTL (**A**, **D**, **G**, **J**), SBA (**B**, **E**, **H**, **K**), or SNA (**C**, **F**, **I**, **L**) and for the PT-marker megalin (LRP2; **A**–**C**), the TAL-marker NKCC2 (**D**–**F**), the DCT-marker NCC (**G**–**I**), or the CNT/CD-marker AQP2 (**J**–**L**). LTL-FITC is always shown in green, while other FITC conjugates are always depicted in cyan. SNA-Cy3 is always shown in magenta, while SBA-Alexa647 is shown in cyan. Protein segment markers stained with Alexa546 or Cy3 are shown in either red or magenta to avoid red-green signals in merged images. For the same reason, protein segment markers stained with Alexa488 or Cy2 are shown in either green or yellow. Single channels depicted in grayscale can be found in Supplementary Figs. 2–4. LTL only overlapped with the PT (**A**), but did not stain the TAL (**D**), DCT (**G**), or CNT/CD (**J**). Strong SBA staining partially colocalized with the DCT (**H**) or CNT/CD (**K**), whereas faint SBA signal was found to stain a PT subpopulation of weakly labeled LRP2-positive tubules (**B**). In contrast, SBA did not stain the TAL (**E**). SNA colocalized with glomeruli and overlapped with the CNT/CD (**L**), while no SNA staining was detected in the PT (**C**), TAL (**F**), or DCT (**I**)

To better characterize the identity of the Flaggs-labeled nephron segments, we performed multiple co-staining by combining each Flagg with one of the four different nephron segment-specific markers LRP2 (PT), NKCC2 (TAL), NCC (DCT), or AQP2 (CNT/CD). In Fig. 3, representative overlay images of all 12 possible combinations are shown with high magnification. Single-channel images with lower magnification are provided in Supplementary Figs. 2–4. LRP2-labeled tubules completely overlapped with LTL (Fig. 3A). Weaker LRP2 staining was partially SBA-positive (Fig. 3B), whereas SNA did not colocalize with the PT-marker (Fig. 3C). NKCC2 staining overlapped with neither LTL, SBA, nor SNA (Fig. 3D–F). The only Flagg that showed a co-stain with NCC-labeled tubules was SBA (Fig. 3H). However, many NCC-positive tubules did not stain for SBA (Fig. 3H and Supplementary Fig. 3G–H). AQP2-positive tubules completely overlapped with SNA (Fig. 3L) and a majority exhibited co-staining with SBA (Fig. 3K), whereas no colocalization was observed for LTL (Fig. 3J). Additionally, we found that SNA always labeled the glomeruli. Based on these findings, we concluded that LTL could be used to sort the PT and that SBA seemed suitable to sort part of the DCT. Furthermore, SNA could be used to sort glomeruli, and a combination of SNA and SBA could enrich the CNT/CD segments. In contrast to the other nephron segments, the TAL could be sorted as a “dark,” non-fluorescent population. Table 2 provides a summary of which nephron structures are marked by which lectin.

**Table 2:** Staining specificity of LTL, SBA, and SNA for mouse or human nephron segments

Lectin/agglutinin	Glom	PT	TAL	DCT	CNT/CD
Lotus tetragonolobus lectin (**LTL)**		ms, hu			
Soybean agglutinin (**SBA**)		hu_P_		ms_P_	ms_P_, hu
*Sambucus nigra* lectin (**SNA**)	ms, hu			hu_P_	ms, hu

LTL, SBA, and SNA binding was assessed on mouse (*ms*) or human (*hu*) kidney cryosections. Glomeruli (*Glom*) were identified by morphology, PT by LRP2 (*megalin*), TAL by NKCC2, DCT by NCC, and CNT/CD by AQP2. No staining (–), partial staining (*P*)

### Conjugation of SBA with Pacific Blue

Most commercially available lectins or agglutinins are only available as FITC, Cy3, Alexa594, or Alexa647 conjugates. Therefore, for our fluorescence staining experiments, we have so far combined fluorophores that can be excited in the green, red, and far-red ranges. However, this combination, especially the simultaneous use of Cy3 and Alexa647, is not optimal for flow sorting on the MoFlo ASTRIOS EQ system, as due to the low system pressure of 5 psi, the first pinhole with the red laser (640 nm) gets out of range of accepted laser delay times. Therefore, Alexa647 cannot be excited with the red laser, but only with the yellow-green laser (561 nm), resulting in a reduced excitation efficiency of about 15%. Overcoming this limitation, we decided to in-house label unconjugated SBA with Pacific Blue (PB), which can be excited with a violet laser. The degree of labeling (DoL) of SBA-PB was estimated to correspond to 2.87 mol dye per mole protein. To evaluate this DoL in comparison to the other SBA conjugates, we stained renal cryosections with either 2, 4, 8, or 16 µg/ml SBA-PB. Applying the same exposure settings, we found the fluorescence intensities to be too strong for the 16, 8, and 4 µg/ml SBA-PB staining solutions (Supplementary Fig. 5). Even 2 µg/ml SBA-PB still gave a rather strong signal intensity. Thus, in order not to overstain the tubules with SBA-PB for tissue sorting, we decided to proceed with 0.5 µg/ml (1.25 µl).

### Flaggs-aided sorting of tubular nephron segments

**Fig. 4:**
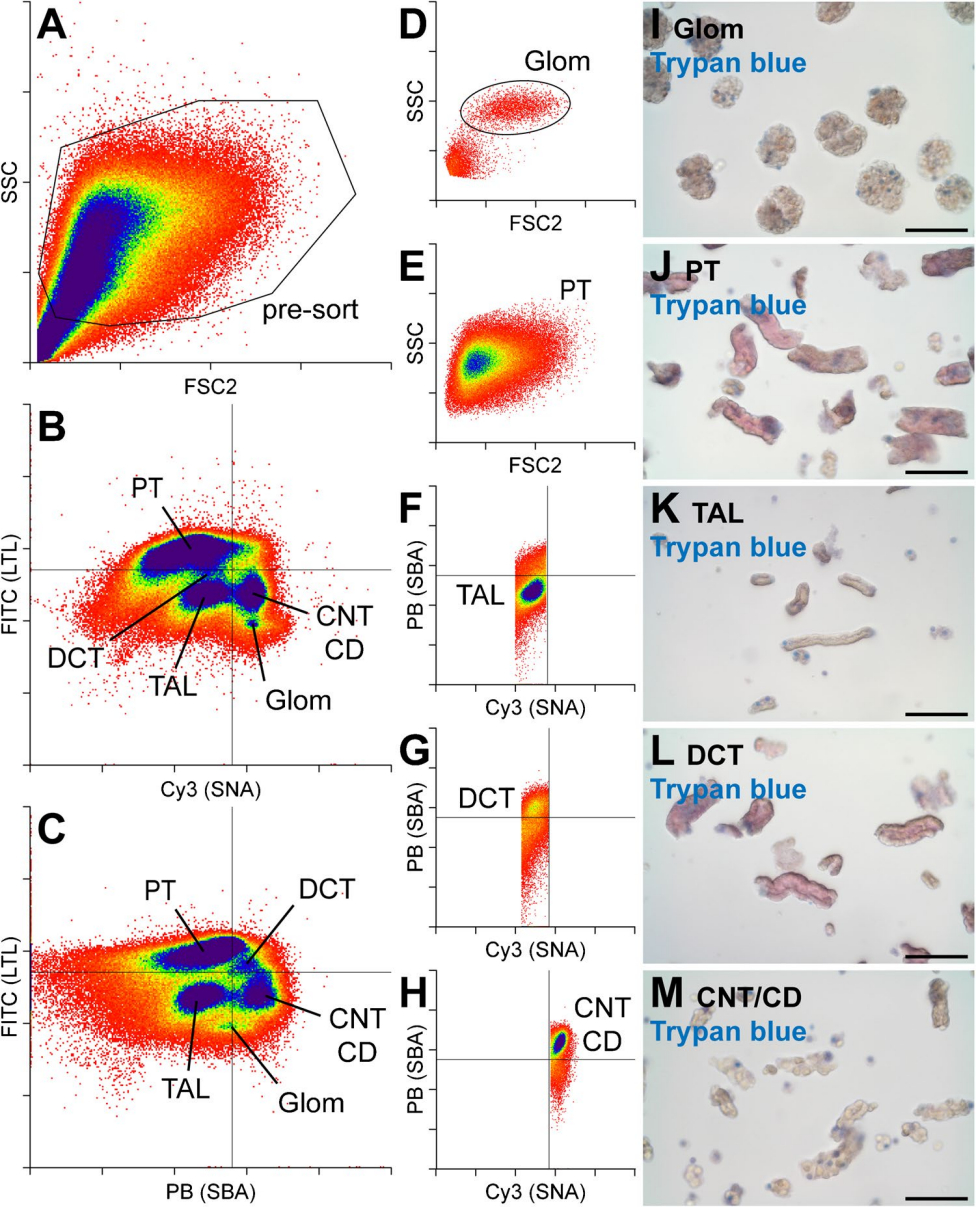
Sorting strategy and viability of sorted nephron segments from mice. Freshly isolated nephron segments were stained with LTL-FITC, SBA-PB, and SNA-Cy3 and subjected to flow sorting. (**A**–**H**) FACS scatterplots, showing the initial separation with forward (FSC2) and sideward (SSC) scattering (**A**) and the gate (pre-sort) that was used for secondary (**B**, **C**) and tertiary separation (**D**–**H**). Secondary separation was based on FITC and either Cy3 (**B**) or PB signal intensity (**C**). The different nephron segment populations are marked in these plots. Tertiary separations were based on SSC/FSC2 for Glom (**D**) and PT (**E**) or PB/Cy3 for TAL (**F**), DCT (**G**), and CNT/CD (**H**). Representative images of the sorted nephron segments are shown in (**I**–**M**). These segments were additionally stained with Trypan blue to assess viability. Scale bar 100 µm

Using LTL-FITC, SNA-Cy3, and our in-house labeled SBA-PB, we established a sorting strategy to separate five distinct nephron segments. First, the tubule fragments were separated according to size (forward scatter—FCS2) and structural differences (sideward scatter—SSC) (Fig. 4A). From this scatter plot, approximately 60% off all events were included in the “pre-sort” gate, excluding single cells and cellular debris that scattered towards the origin of the plot. In the next step, we separated the “pre-sort”-gated segments based on FITC and Cy3 (Fig. 4B) or FITC and PB signal intensities (Fig. 4C). Already on these secondary scatter plots, five distinct clusters, corresponding to PT, TAL, DCT, CNT/CD, and glomeruli (Glom), can be distinguished. SNA-positive Glom were found in the lower right quadrant of the FITC/Cy3 scatter (Fig. 4B), whereas LTL-positive PT segments clustered to the upper left quadrants in both scatter plots. TAL segments were in the lower left quadrants of both scatter plots, where unstained segments were expected, and CNT/CD segments were enriched in the lower right quadrants, indicating dual SNA and SBA positivity, but a lack of LTL binding. To our surprise, DCT segments accumulated in the upper right quadrant of the FITC/PB scatterplot, where double-positive LTL/SBA tubules should be found (Fig. 4C). However, we did not observe such a pattern in our staining experiments on kidney sections (Fig. 1D–F). Since the quality of the green signal of the DCT population was different from the LTL-FITC-specific signal and of lower intensity, we concluded that the sorting system detected a second signal in the green channel that was independent of LTL-FITC. Most likely, this corresponds to a DCT-specific background signal. Based on these distribution characteristics of the nephron segments along the FITC/Cy3 or FITC/PB axes, the final sorting gates were established (Fig. 4D–H). Since Glom or PT populations were only positive for SNA-Cy3 or LTL-FITC, respectively, both clusters were once more separated using FSC2/SSC. For the glomeruli, two populations emerged, of which the upper one, which corresponded to larger and structurally more complex structures, contained only glomeruli (Fig. 4D). In contrast, the PT segments, which appeared more homogeneous, were collected as one population (Fig. 4E). TAL, DCT, and CNT/CD segments were gated again using PB and Cy3 signal intensities. The TAL segments were finally sorted from the lower left (double negative) quadrant of this tertiary scatterplot (Fig. 4F). Conversely, DCT tubules were collected from the upper left (PB-positive, Cy3-negative) quadrant (Fig. 4G), whereas CNT/CD segments were sorted from the upper right (double positive) quadrant (Fig. 4H and M). The sorted nephron segments showed different morphologies (Fig. 4I–M). Glom were of course round and cloud-like structures (Fig. 4I). The PT and DCT segments were large tubules with smooth surfaces (Fig. 4J and L ), whereas TAL segments were much thinner tubules (Fig. 4K). CNT/CD segments had a cobblestone-like surface from which individual cells protruded (Fig. 4M). Depending on the dilution of the nephron segment suspension, between 1400 and 2200 events were analyzed per second. Details on the percentage distribution of the different nephron segments within this flow and the number of nephron segments sorted per second are shown in Table 3. Overall, from kidney collection to the end of sorting, the entire procedure took approximately 3 h, of which the sorting required 45 to 75 min.

**Table 3:** Sorting characteristics and yield for mouse nephron segments

	Glom	PT	TAL	DCT	CNT/CD
Gated from “pre-sort” (%)	0.84 ± 0.35	29.60 ± 11.17	10.73 ± 2.62	2.19 ± 0.70	5.96 ± 1.29
Sorted events/s	6 ± 2	175 ± 55	65 ± 16	15 ± 5	36 ± 8
Protein/event (pg)	nd	2858 ± 1024	534 ± 130	2863 ± 324	849 ± 110
Protein yield/s (ng)	nd	521 ± 213	45 ± 16	54 ± 21	37 ± 7
Time for 10 µg protein (min:sec)	**-**	**0:19**	**3:42**	**3:05**	**4:30**
*RNA/event (pg)*	*159* ± *88*	*131* ± *84*	*24* ± *7*	*118* ± *37*	*45* ± *16*
*RNA yield/s (ng)*	*0.71* ± *0.33*	*16.71* ± *5.83*	*1.77* ± *0.47*	*1.81* ± *0.74*	*1.52* ± *0.44*
Time for 1 µg RNA (min:sec)	***23:38***	***1:00***	***9:25***	***9:12***	***10:59***

The first two rows give details on the gated and sorted events for each nephron segment. Rows 3–5 inform about the amount of protein that can be recovered in a given time, whereas rows 6–8 are dedicated to RNA yield. Not determined (*nd*)

### Characterization of sorted nephron segments

To assess tubule viability after sorting, we performed Trypan blue staining on freshly sorted tubules (Fig. 4I–M and Supplementary Fig. 6). Trypan blue is not taken up by viable cells; however, it can cross the membrane of dead cells, which then exhibit a distinctive blue coloration. Overall, the number of dead cells was very low in all sorted nephron segments, thus indicating that the tissue sorting process is compatible with tubule segment vitality and does not cause gross damage to the tubules. We also determined the protein and mRNA yield that can be recovered for each tubular nephron segment upon sorting (Table 3). For example, the protein yield per second of the PT was 521 ng, which was tenfold higher compared to all other segments.

**Fig. 5:**
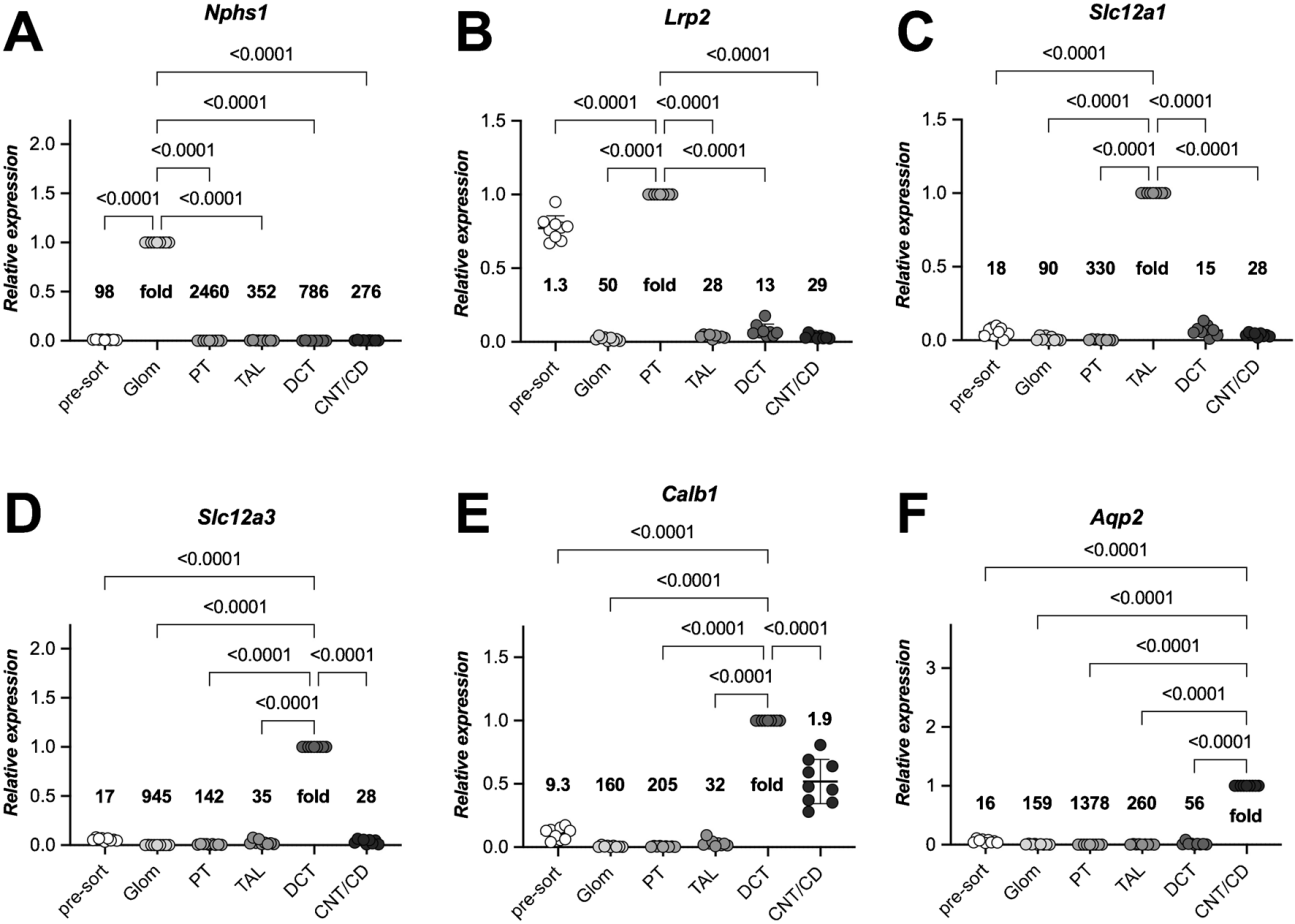
Validation of sorted nephron segments from mice by mRNA expression levels. Kidneys of nine mice were subjected to our sorting procedure, and the identities of the five sorted nephron segments were assessed by determining the mRNA expression levels of segment-specific (**A**–**D**, **F**) and a segment-overlapping marker (**E**) and compared to pre-sort samples. The expression level of each marker was normalized to the segment for which the highest value was expected (i.e., *Nphs1* (**A**) for Glom, *Lrp2* (**B**) for PT, *Slc12a1* (**C**) for TAL, *Slc12a3* (**D**) for DCT, *Calb1* (**E**) for DCT and CNT/CD, and *Aqp2* (**F**) for CNT/CD). In addition, the x-fold **change** for each marker in relation to the other nephron segments is shown for each diagram. Ordinary one-way ANOVA with Dunnett correction for multiple comparisons

In addition to these parameters, we also validated the identity and purity of the sorted nephron segments by assessing the mRNA expression levels of a panel of 11 segment-specific and four segment-overlapping markers (Fig. 5 and Supplementary Fig. 7). For better comparability of results among different mice, the expression levels of each marker were normalized to the segment for which the highest value was expected. From this, we then calculated the fold enrichment in comparison to the pre-sort and all other sorted segment samples. The Glom marker nephrin (*Nphs1*) was 98-fold enriched in the Glom population compared to the pre-sort samples and more than 2000-fold in comparison to the PT (Fig. 5A). These extraordinary high enrichment scores are probably due to the non-tubular structure of the glomerulus that allows a better separation based on forward and sideward scatters (Fig. 4D) than is the case for the separation of different tubule segments. Megalin (*Lrp2*), a specific marker of the PT, was found to be enriched 1.3-fold compared to before sorting (Fig. 5B). This may be attributed to the fact that the PT constitutes the majority of all tubular fragments in the presorting samples. On the other hand, *Lrp2* expression in the PT was 13- to 50-fold higher compared to all other sorted nephron segments, which still indicates a very good sorting efficiency. The TAL marker *Slc12a1* (Fig. 5C), the DCT marker *Slc12a3* (Fig. 5D), or the CNT/CD marker *Aqp2* (Fig. 5F) were enriched in their respective nephron segments 15- to 330-, 17- to 945-, or 16- to 1378-fold, respectively. The expression levels of *Calb1*, a segment-overlapping marker with higher expression levels in the DCT and lower expression levels in the CNT/CD [22], were indeed 1.9-fold higher in our sorted DCT than in the sorted CNT/CD segments (Fig. 5F). Taken together, these results provide strong evidence that our sorting protocol delivers viable nephron segments with high yield and purity.

### Sorting of human samples

**Fig. 6:**
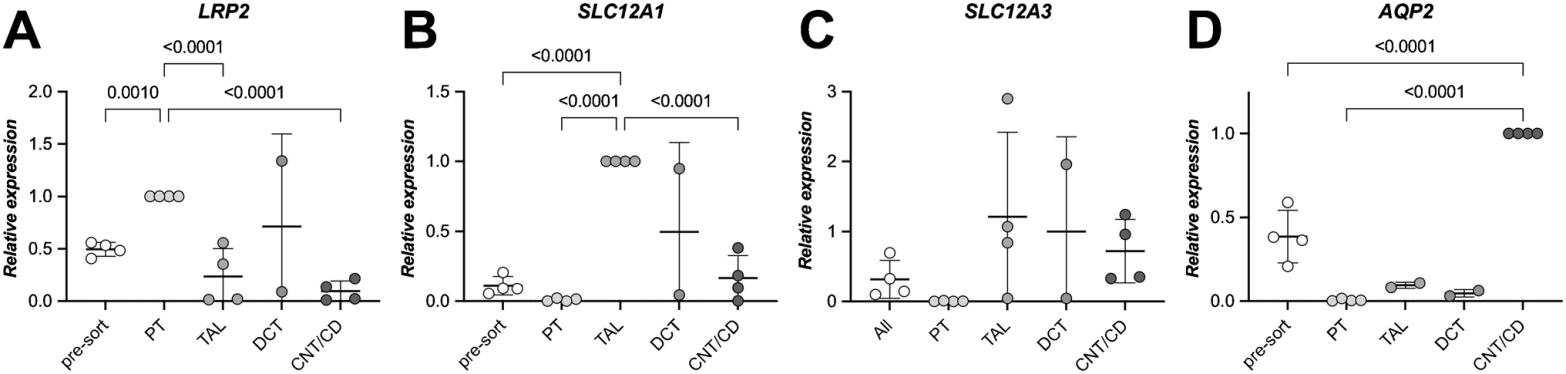
Validation of sorted nephron segments from human samples. Four human kidneys samples were subjected to our sorting procedure, and the identities of four sorted nephron segments were assessed by mRNA expression levels of segment-specific markers. The expression level of each marker was normalized to the segment for which the highest value was expected (i.e., *LRP2* (**A**) for PT, *SLC12A1* (**B**) for TAL, *SLC12A3* (**C**) for DCT, and *AQP2* (**D**) for CNT/CD). A pure DCT segment population could not be sorted. However, a clear enrichment was apparent for PT, TAL, and CNT/CD nephron segments. Ordinary one-way ANOVA with Dunnett correction for multiple comparisons

As the next step, to assess whether our sorting protocol would work for other mammalian species, we applied it to human kidney samples. These human samples, corresponding in size to approximately two adult mouse kidneys, were collected from the apparently healthy portion of nephrectomized kidneys of four male patients (Hu1–Hu4; > 60 years old) with renal cell carcinoma. Compared to mouse kidneys, the human kidney samples were significantly older and much harder, which is most likely due to their higher proportion of connective tissue. Therefore, we increased the dosage of digestive enzymes in the digestive solution. However, despite this adaptation, we obtained fewer and smaller tubule segments from human tissue compared to mouse preparations. Nevertheless, we sorted the human nephron segments (Supplementary Fig. 8) using the gating strategy established in mice. Validation of the identity and purity of the sorted human nephron segments by RT-qPCR (Fig. 6 and Supplementary Fig. 9) revealed that PT, TAL, and CNT/CD were successfully sorted using our protocol. However, we did not obtain a pure DCT population (Fig. 6C and Supplementary Figs. 9C and D). Most glomeruli could not be sorted, since, because of their large size, they were sieved out by the 100-µm cell strainer.

**Fig. 7:**
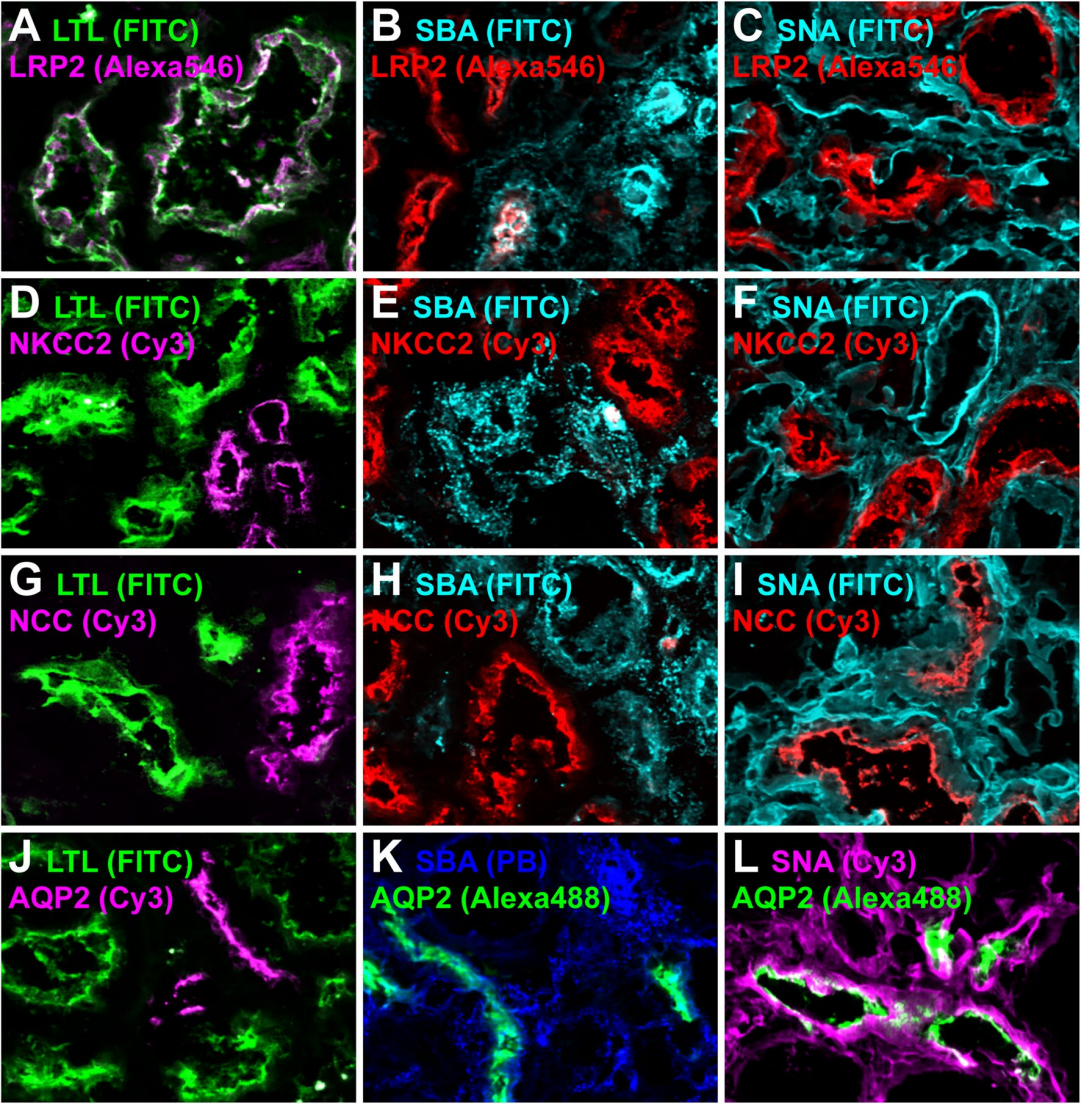
Co-staining of Flaggs and nephron markers on human kidney cryosections. Cryosections were stained with LTL (**A**, **D**, **G**, **J**), SBA (**B**, **E**, **H**, **K**), or SNA (**C**, **F**, **I**, **L**) and for the PT-marker megalin (LRP2; **A**–**C**), the TAL-marker NKCC2 (**D**–**F**), the DCT-marker NCC (**G**–**I**), or the CNT/CD-marker AQP2 (**J**–**L**). LTL-FITC is always shown in green, while other FITC conjugates are always depicted in cyan. SNA-Cy3 is always shown in magenta, while SBA-PB is shown in blue. Protein segment markers stained with Alexa546 or Cy3 are shown in either red or magenta to avoid red-green signals in merged images. AQP2-Alexa488 is only shown in green. Single channels depicted in grayscale can be found in Supplementary Figs. 10–12. LTL only overlapped with the PT (**A**) but did not stain the TAL (**D**), DCT (**G**), or CNT/CD (**J**). SBA staining partially colocalized with the PT (**B**) and the CNT/CD (**K**) but not with the TAL (**E**). SNA colocalized with glomeruli and overlapped with the CNT/CD (**L**), while no SNA staining was detected in the TAL (**F**). However, unlike mice, the DCT on human sections was not stained by SBA (**H**) but instead showed a weak SNA signal (**I**)

To get an idea why we could not sort human DCT segments, we went back a step and performed fluorescent double staining with Flaggs and segment-specific markers on fresh-frozen human kidney sections. In Fig. 7, representative overlay images of all 12 possible combinations are shown with high magnification. Single-channel images with lower magnification are provided in Supplementary Figs. 10–12. Similar to mice (Fig. 3A–F), the PT marker LRP2 overlapped completely with LTL (Fig. 7A), and only partially with SBA (Fig. 7B), but not with SNA (Fig. 7C), while the TAL marker NKCC2 did not show any overlap with LTL, SBA, or SNA (Fig. 7D–F). For AQP2, a marker of the CNT/CD, no overlap with LTL was detected (Fig. 7J), but complete co-staining of AQP2 was evident with SBA and SNA (Fig. 7K, L), which was largely comparable to mice (Fig. 3J–L). Human DCT tubules, labeled with NCC, were, as in mice, LTL negative (Figs. 3G and 7G). However, in stark contrast to mice (Fig. 3H), these nephron segments did not overlap with SBA (Fig. 7H) but showed weak SNA positivity, at least in Hu4 (Fig. 7I). Therefore, based on these findings, we will adapt the sorting protocol for human samples, especially for DCT segments, which should be separable by gating FITC-negative, SBA-negative, but SNA-positive events.

## Discussion

In the present study, we established a cost-efficient lectin/agglutinin-aided sorting protocol for the simultaneous dissection of the five main nephron segments (Glom, PT, TAL, DCT, and CNT/CD) from the same kidney with high material yield. While searching for a suitable combination of Flaggs that would allow the separation of several nephron segments, we realized which profound impact tissue pretreatment has on Flaggs binding specificity. In our hands, formalin-fixed paraffin-embedded (FFPE) tissue was not a good representation of the Flaggs binding pattern in native, unfixed tissue. Further attention should be paid to the high binding affinity of certain Flaggs to BSA or proteins of the extracellular matrix, especially collagens [36] that might distort the staining specificity. Other difficulties we faced in setting up the procedure included replacing a fluorophore to ensure compatibility of all lasers under the low-pressure settings required to sort the large nephron segments. Our method surpasses classical sorting methods (conventional manual microdissection or flow sorting of single cells) on multiple levels. (1) In superior efficiency yield, we do not sort single cells, but intact tubular nephron segments, some of considerable length (> 100 µm), consisting of dozens or hundreds of epithelial cells in their native arrangement. Thus, within minutes, a multiple of biomaterial can be collected that normally would require hours of sorting (Table 3). (2) In cheaper labeling procedure, the use of lectins for labeling and sorting offers an inexpensive alternative over antibody-mediated approaches as 1 mg of an already labeled lectin costs 10- to 20-fold less than the equal amount of an antibody suitable for flow cytometry, excluding the expense for additional secondary antibodies. (3) On user friendliness, with access to a FACS facility, every researcher (especially those without previous microdissection experience) can easily and unbiasedly obtain tubular nephron segments. (4) On broad applicability, our method is principally suited to sort nephron segments from wild-type or genetically modified mice of all ages, human patient biopsies, or other mammalian species. For the latter, the Flaggs combination or gating strategy will most likely need to be adjusted to account for species-related differences [8, 9, 21, 39]. Already in 1983, Schulte and Spicer described species-specific differences in the kidney [31]. They and others used horseradish peroxidase conjugates to compare the lectin binding pattern on histological paraffin section of mouse and rat kidneys [11, 21, 31]. In our hands, a more in-depth characterization of the Flaggs PNA and DBA, which were excluded from this study for formal reasons, could be useful. This also applies when individual nephron segments are to be sorted into sub-segments (e.g., convoluted and straight PT segments) or for a clean separation of CNT and CD. In the clinic, the availability and molecular characterization of such individual nephron segments or subsegments could help to address unmet medical needs in delayed graft function [5] or toxic nephropathies [27], which could help to better guide clinical management and to define safe therapy endpoints with the aim to reduce the risk of organ rejection or loss of renal function, respectively. However, while highly desirable, a clinical application of our method using surveillance biopsies is at this stage very speculative. On the other hand, our method could be immediately used to assess sex-specific differences in individual nephron segments using proteomics or other omic approaches. When evaluating such studies, a possible influence on the functional properties of the sorted nephron segments by the bound Flaggs themselves should always be kept in mind, since various plant lectins are known to have toxic effects in animals, both locally in the digestive tract and systemically [42]. These harmful effects include mitogenic stimulation of different organs, induction of apoptosis and autophagy, and immunomodulatory functions [18]. For example, SBA was described to dose-dependently increase reactive oxygen species in HeLa cells [24], inhibit plasma membrane repair of damaged gut epithelial cells [19], or induce apoptosis in the naïve porcine jejunal cell line IPEC‐J2 through mitochondrial and ER stress pathways and the downregulation of cytoskeleton proteins [23]. Yet, in comparison to these studies, we do not expect such effects with our method, which is due to the low concentration of Flaggs (1–2 µg/ml vs. 20–2000 µg/ml), the low temperature during incubation and sorting (4 °C vs. 37 °C), and finally, the relatively short time frame (less than 2 h vs. 24-h incubation periods). As another important goal, we want to characterize in more detail how long the sorted segments retain functional properties, in addition to their preserved morphology. To this end, we plan to use our freshly sorted nephron segments as building blocks for drop-on-demand bioprinting processes that enable their precise spatial deposition into 3D hydrogel materials [26, 40]. Technically, this should be feasible and easily achievable since common bioprinting protocols already make use of highly concentrated cell suspension (2 × 10^7^ cells/ml) and 200-µm capillary nozzle diameters [40], which corresponds exactly to the nozzle size we used for sorting. The main advantage of using sorted nephron segments for bioprinting over cell suspensions is that they are already tubular structures with an intact lumen and correct cell polarization, whereas cell suspensions require at least 7 days in culture to assemble into tubular structures, form a lumen, and polarize normally. Furthermore, given the correct printing sequence, tubule systems could be constructed that replicate the tubular portion of a nephron ex vivo and are quickly available for follow-up experiments. In conclusion, our Flaggs sorting protocol is a cheap, broadly applicable method to recover high yields of pure nephron segments with intact morphology in a relatively short time. It has the potential to replace the laborious manual microdissection or expensive antibody-mediated flow sorting of single cells. Furthermore, in combination with novel 3D culture methods, it might become an important ex vivo research tool that both complies with 3R guidelines in preclinical research and can be used for numerous applications.

### Supplementary Information

Below is the link to the electronic supplementary material.

Supplementary file1 (DOCX 19165 KB)

Supplementary file2 (XLSX 22 KB)

## Data Availability

Data supporting the findings of this study are available from the corresponding author on request. Source data are provided with this paper (Supplementary File 1).
