# Supplementary material for: Lectin-mediated, time-efficient, and high-yield sorting of different morphologically intact nephron segments
Source: Pflugers Arch. 2023 Dec 13;476(3):379–93. doi: 10.1007/s00424-023-02894-w (PMC10847228; doi:10.1007/s00424-023-02894-w)
Supplement: Supplementary file 1 — Supplementary file1 (DOCX 19165 KB) [file 424_2023_2894_MOESM1_ESM.docx]

**Supplementary information for**

**Lectin-mediated, time-efficient and high-yield sorting of different morphologically intact nephron segments**

Authors and affiliations:

**Jessica Roskosch^1^, Uyen Huynh-Do^1,†^, and Stefan Rudloff^1,†,*^**

^1^Division of Nephrology and Hypertension, University of Bern and University Hospital Bern, Freiburgstrasse 15, CH-3010 Bern, Switzerland

†Authors contributed equally to this work.

*Corresponding author: Stefan Rudloff: stefan.rudloff@dbmr.unibe.ch

Tel.: +41 31 632 98 17

Fax: +41 31 632 97 34

**Supplementary Material**

Supplementary Fig. 1. Co-staining of different Flaggs on mouse kidney sections.

Supplementary Fig. 2. Single channels of LTL and nephron markers for mice.

Supplementary Fig. 3. Single channels of SBA and nephron markers for mice.

Supplementary Fig. 4. Single channels of SNA and nephron markers for mice.

Supplementary Fig. 5. Validation of SBA-PB for staining on mouse kidney sections.

Supplementary Fig. 6. Trypan blue staining of mouse nephron segments.

Supplementary Fig. 7. mRNA validation of sorted mouse nephron segments.

Supplementary Fig. 8. Human flow sort scatter plots.

Supplementary Fig. 9. mRNA validation of sorted human nephron segments. Supplementary Fig. 10. Single channels of LTL and nephron markers for human. Supplementary Fig. 11. Single channels of SBA and nephron markers for human.

Supplementary Fig. 12. Single channels of SNA and nephron markers for human.

Supplementary Table 1. Lectins used in this study.

Supplementary Table 2. Primers used for qPCR.

Supplementary File F1 Source data.

**Supplementary Figures**


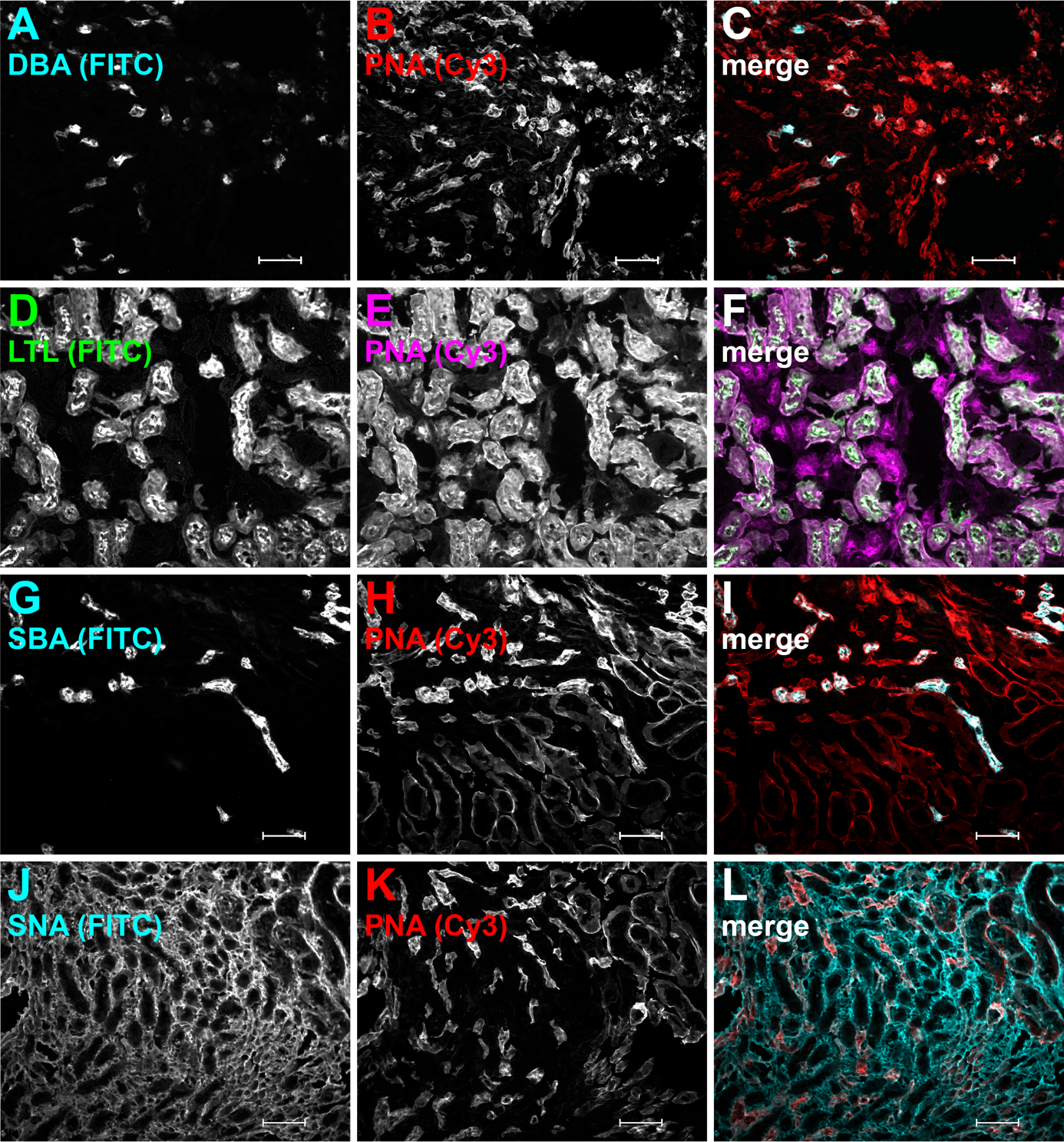


**Supplementary Fig. 1**. Co-staining of different Flaggs on mouse kidney sections.


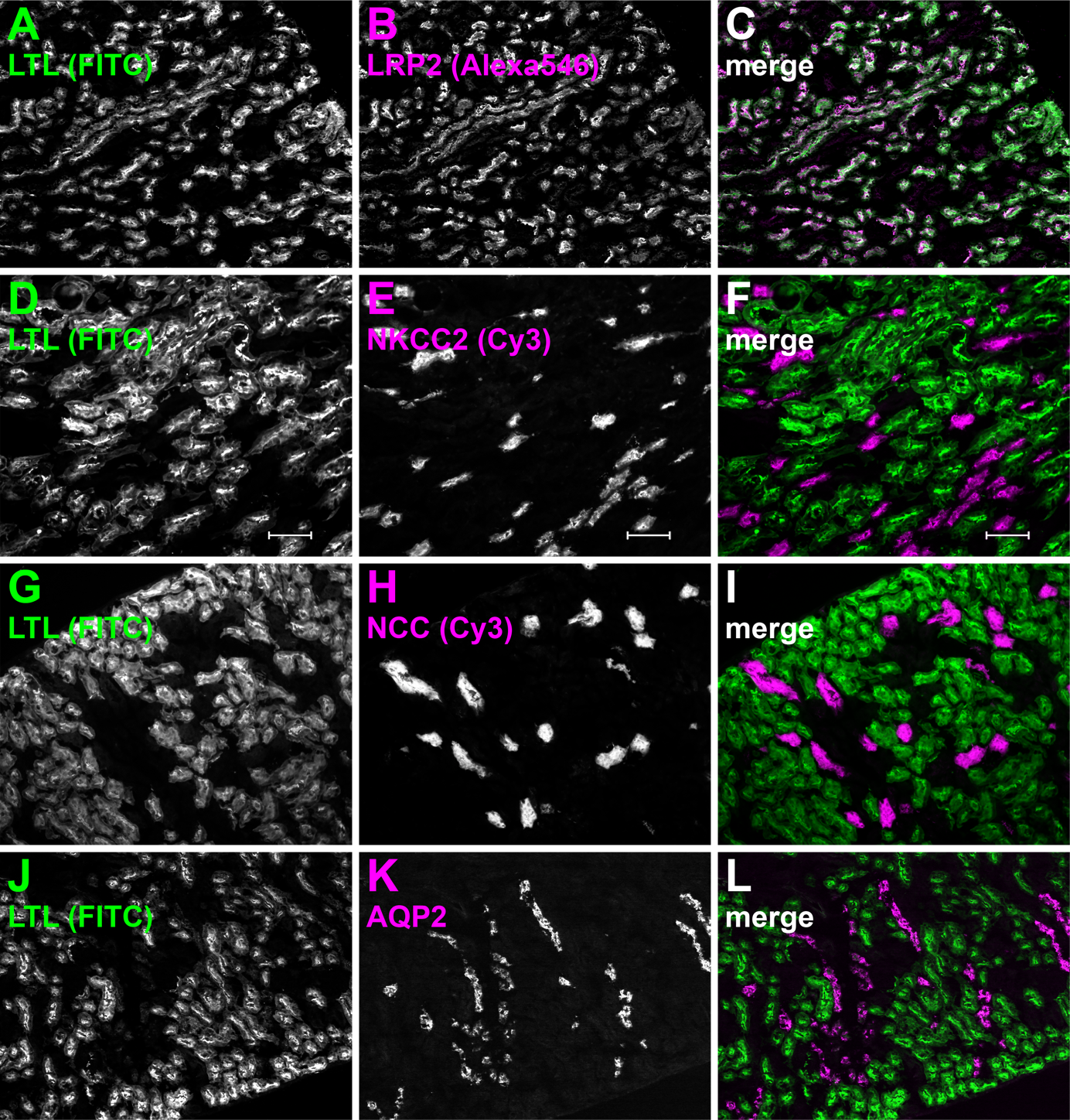


**Supplementary Fig. 2**. Single channels of LTL and nephron markers for mice.


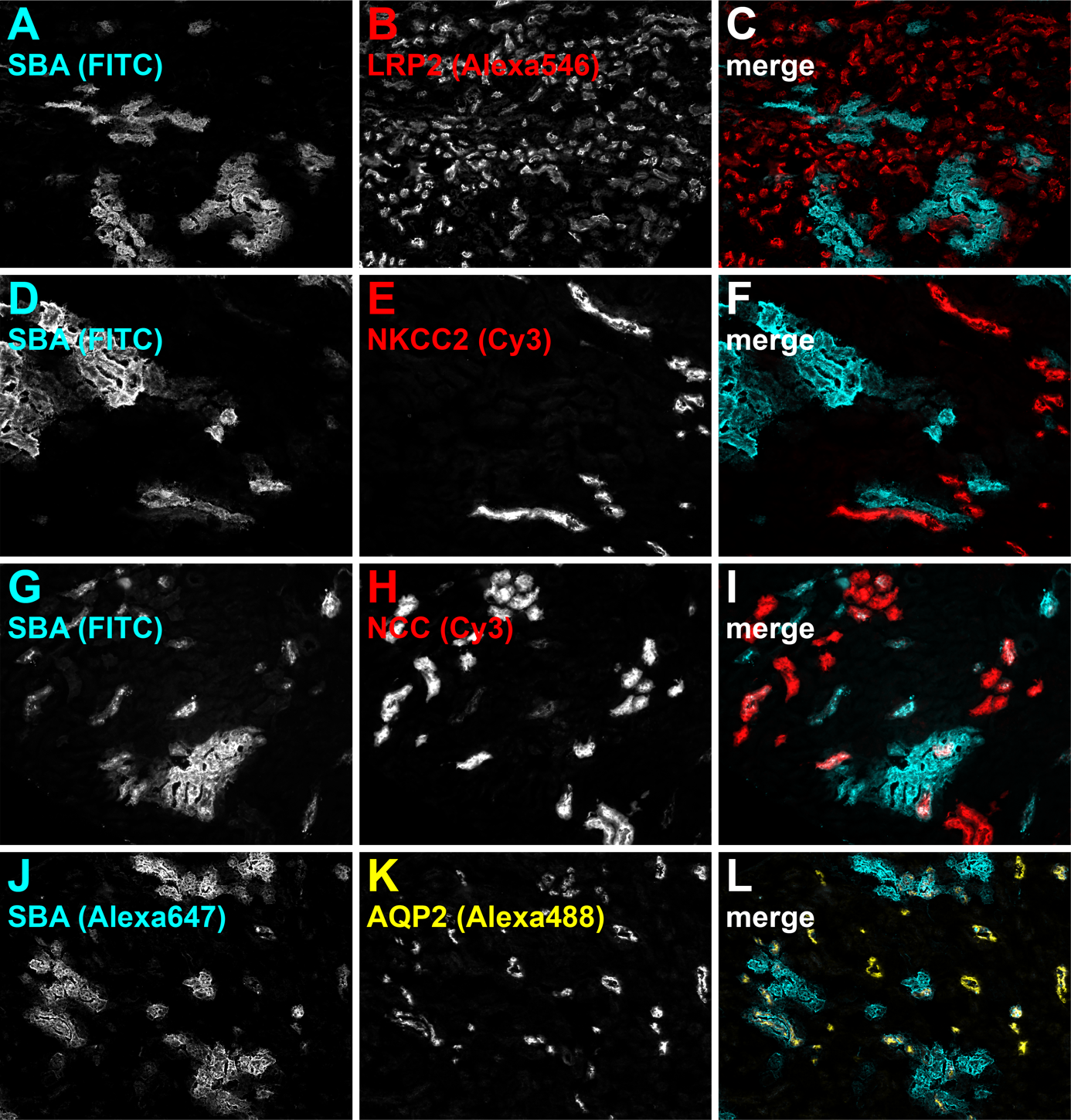


**Supplementary Fig. 3**. Single channels of SBA and nephron markers for mice.


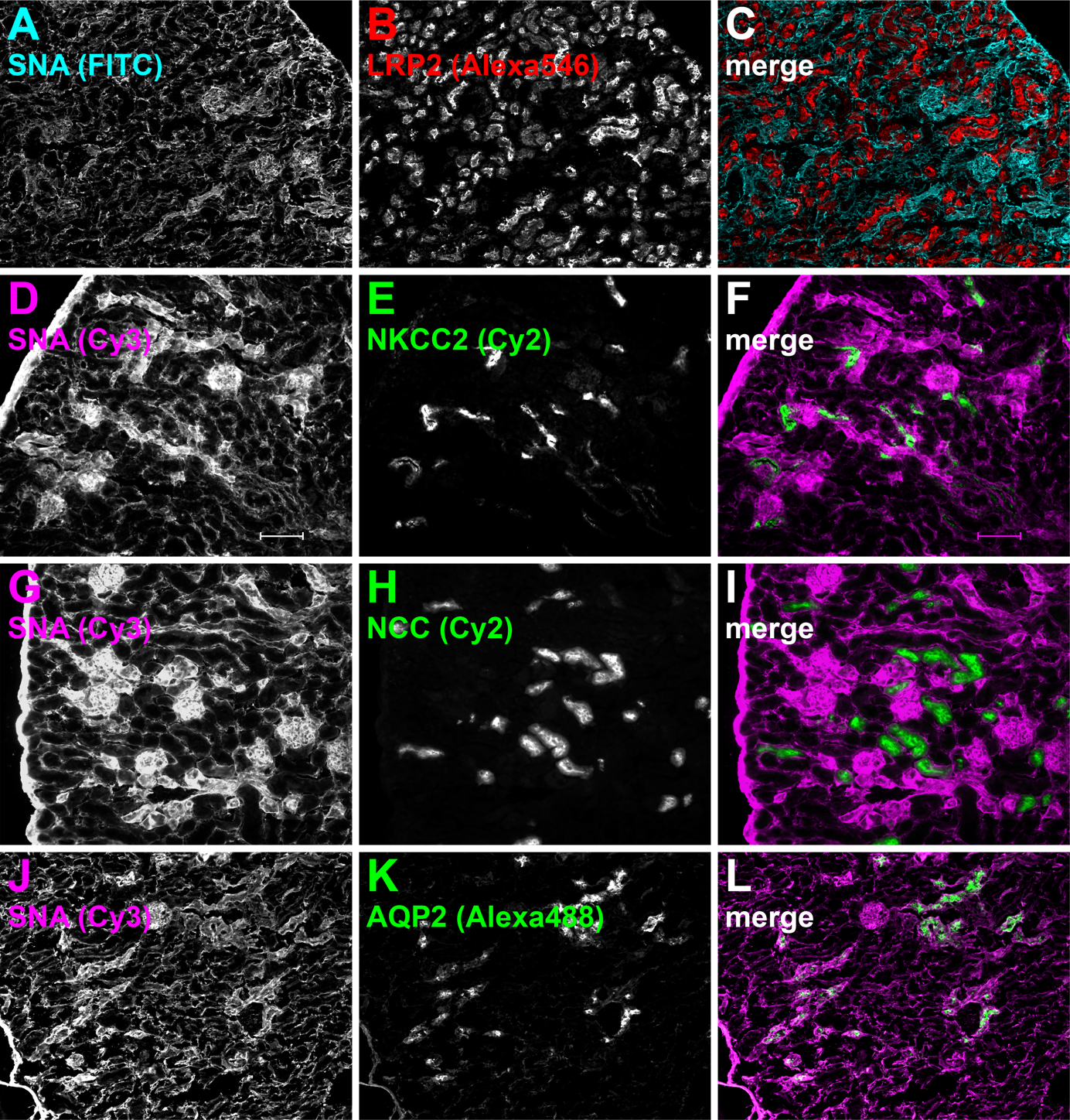


**Supplementary Fig. 4**. Single channels of SNA and nephron markers for mice.


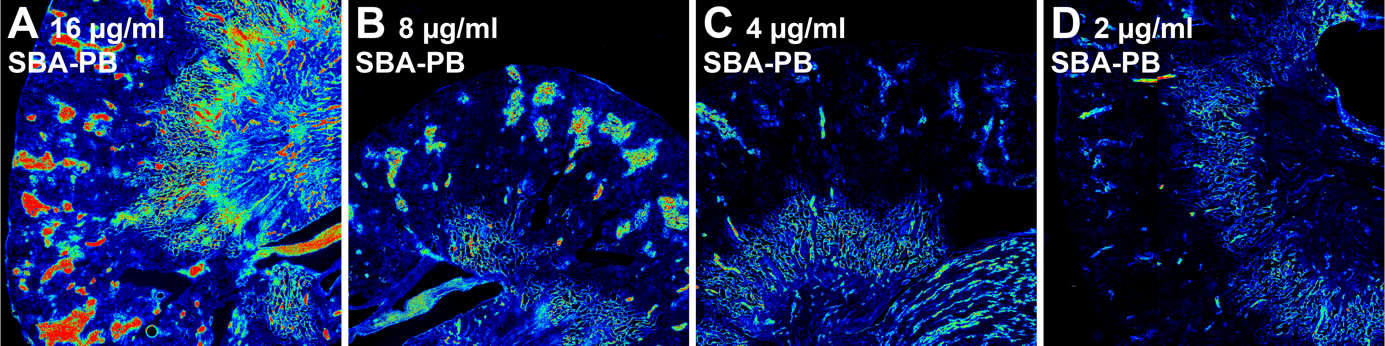


**Supplementary Fig. 5**. Validation of SBA-PB for staining.


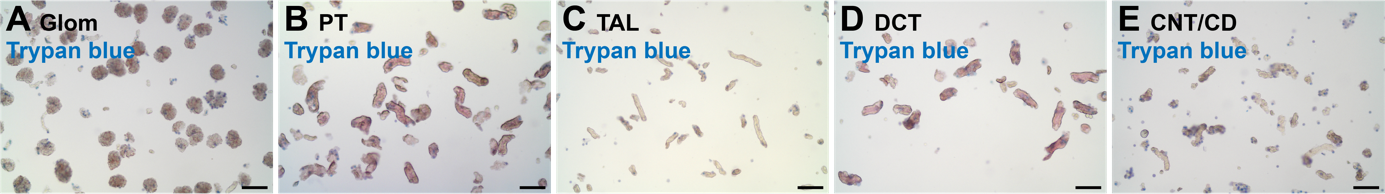


**Supplementary Fig. 6**. Trypan blue staining of mouse nephron segments.


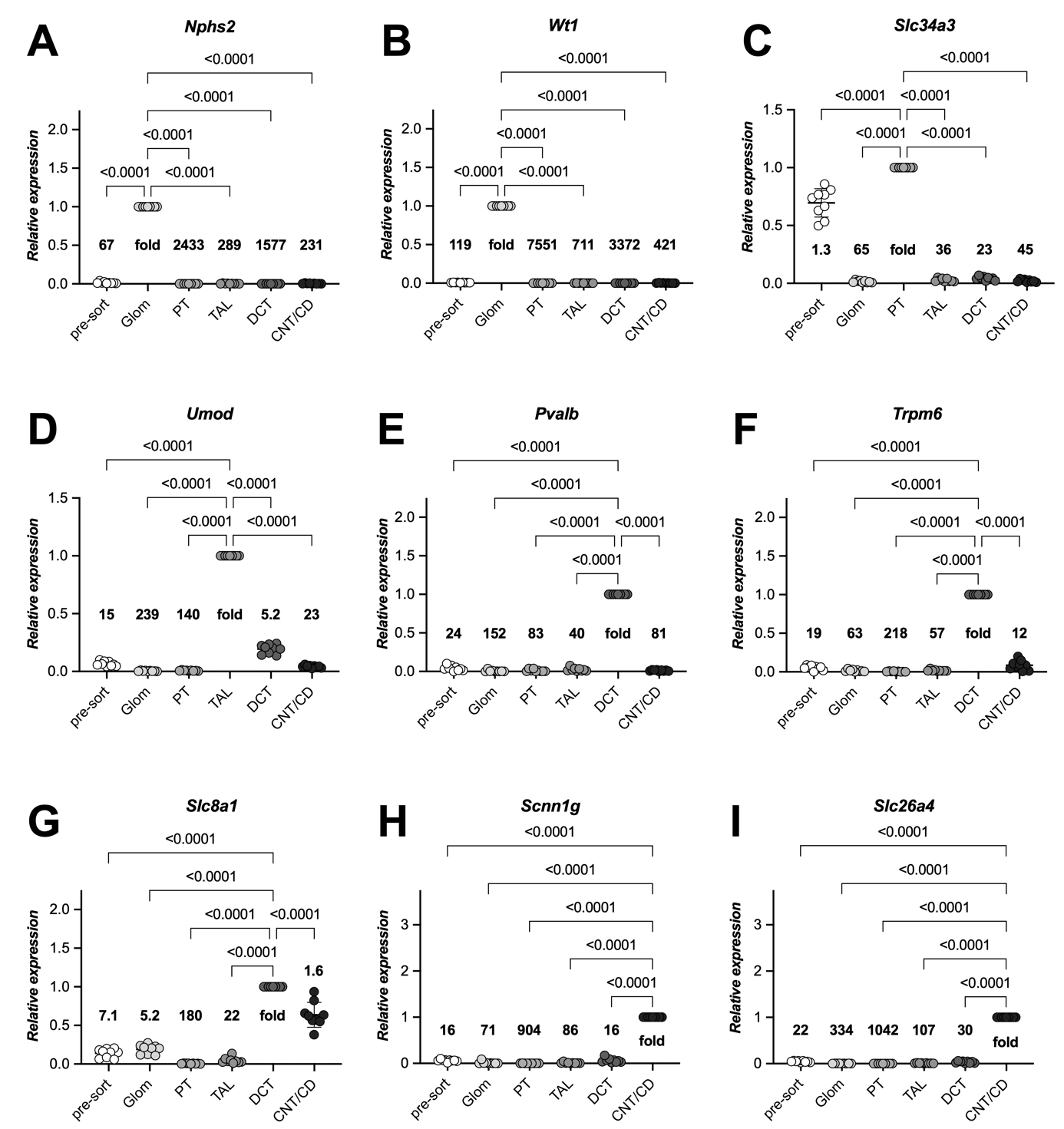


**Supplementary Fig. 7**. mRNA validation of sorted mouse nephron segments.


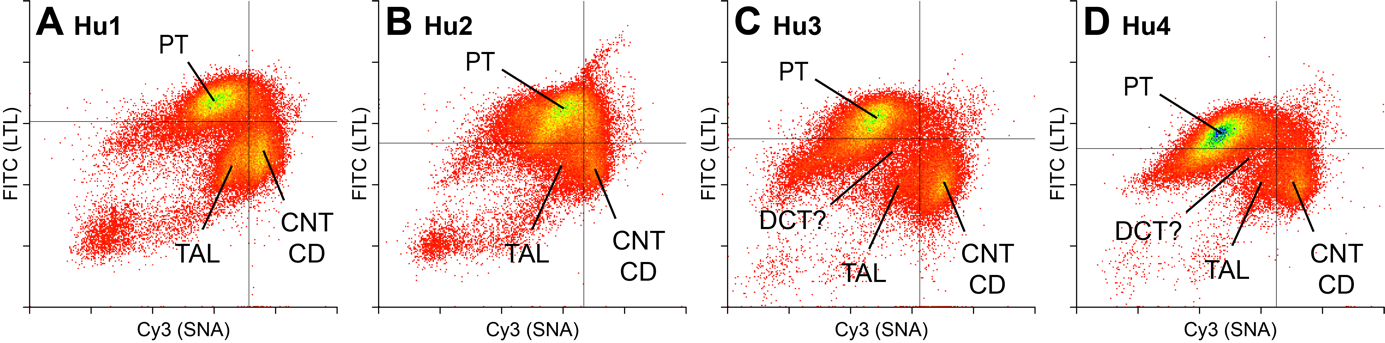


**Supplementary Fig. 8**. Human flow sort scatter plots.


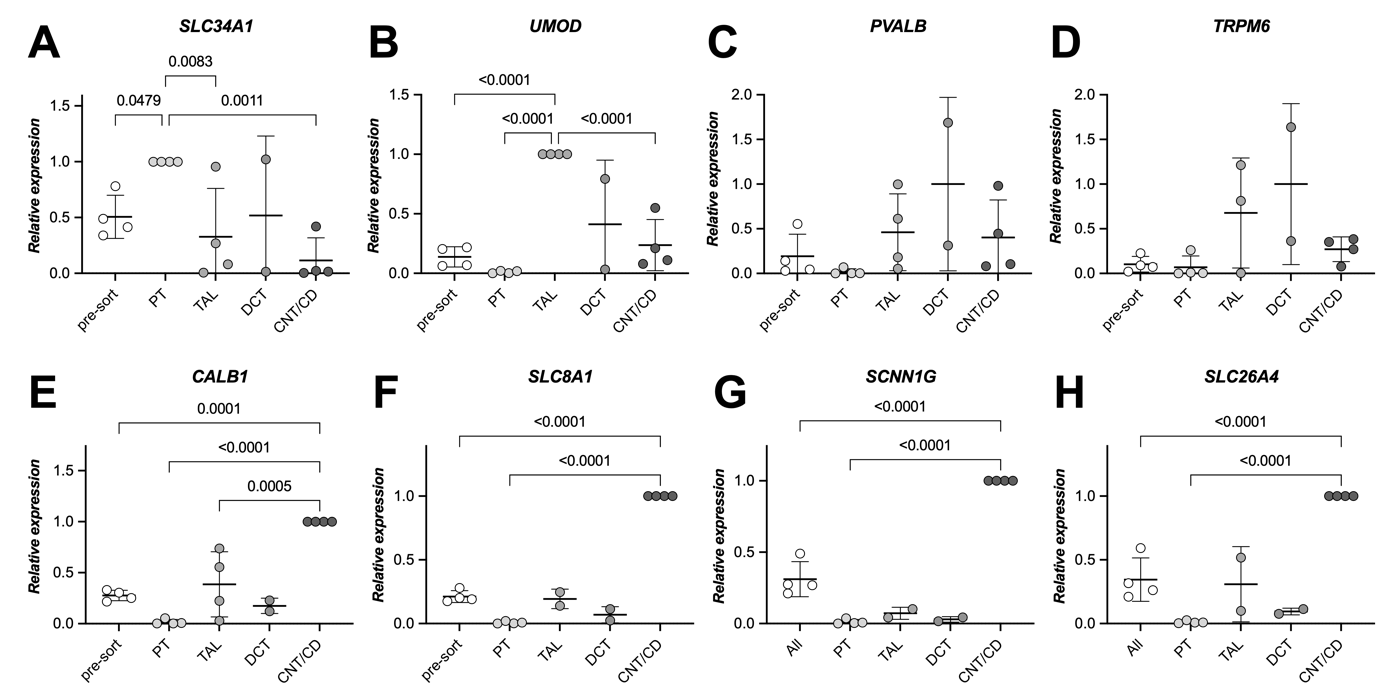


**Supplementary Fig. 9**. mRNA validation of sorted human nephron segments.


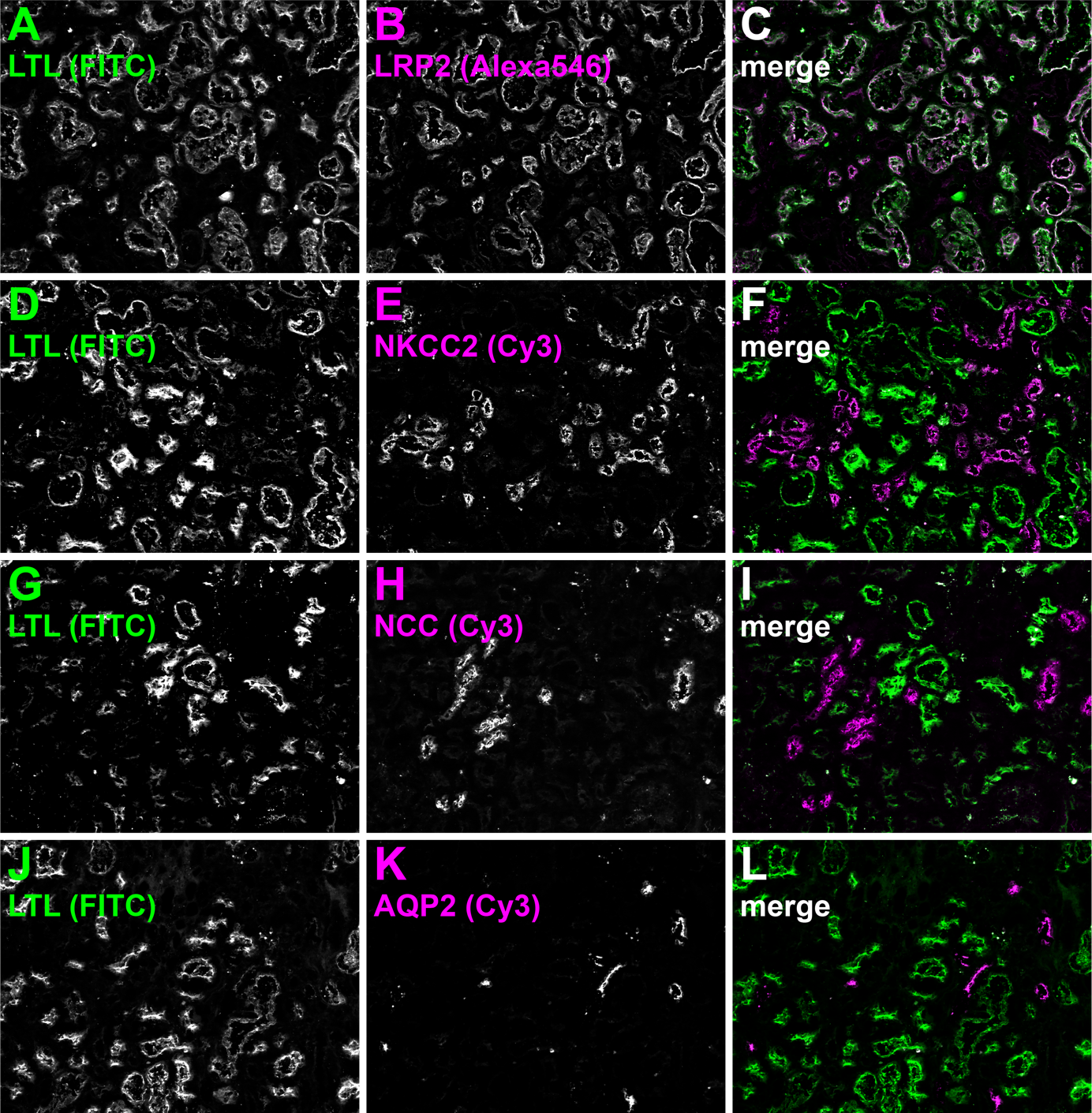


**Supplementary Fig. 10**. Single channels of LTL and nephron markers for human.


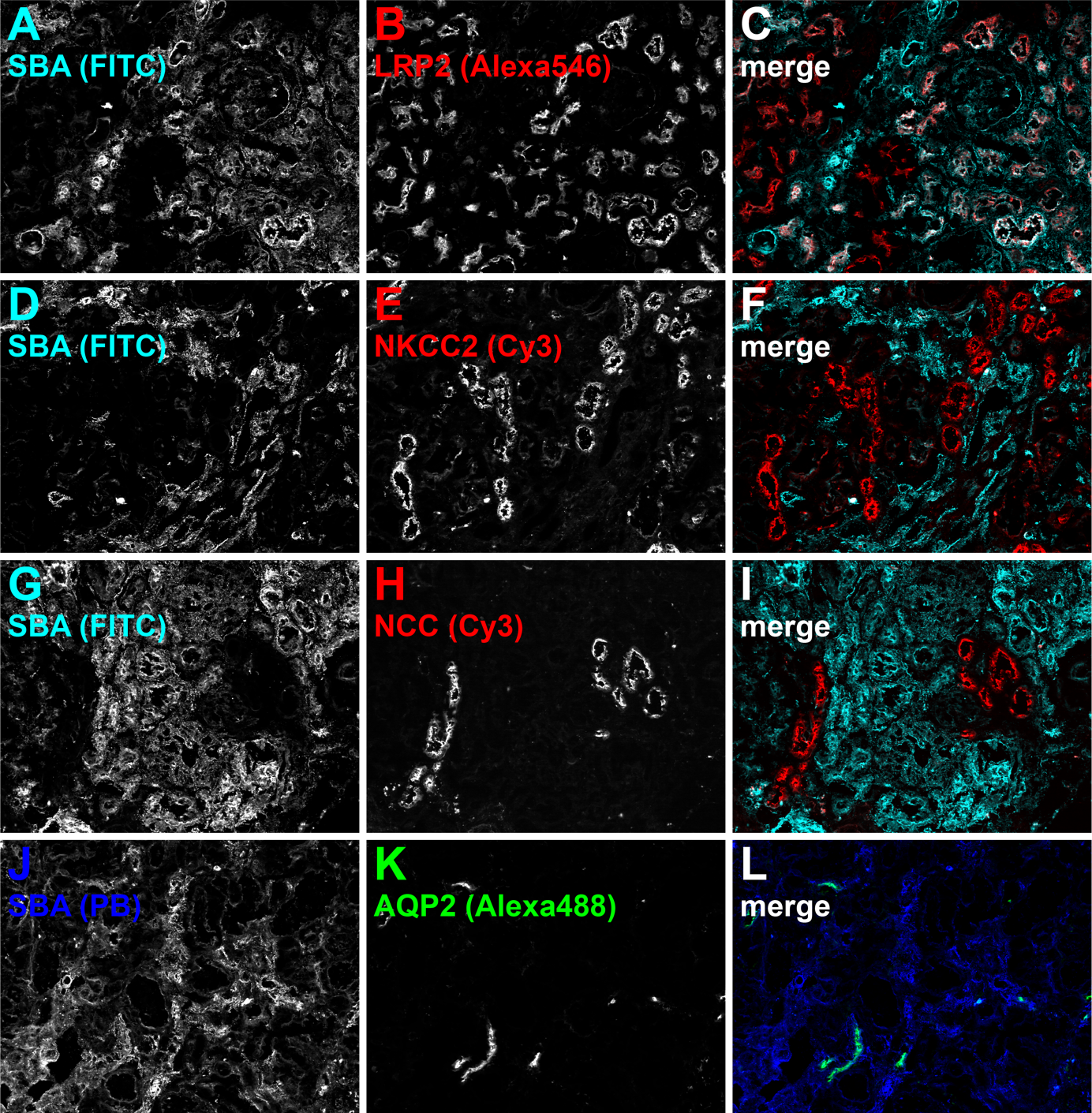


**Supplementary Fig. 11**. Single channels of SBA and nephron markers for human.


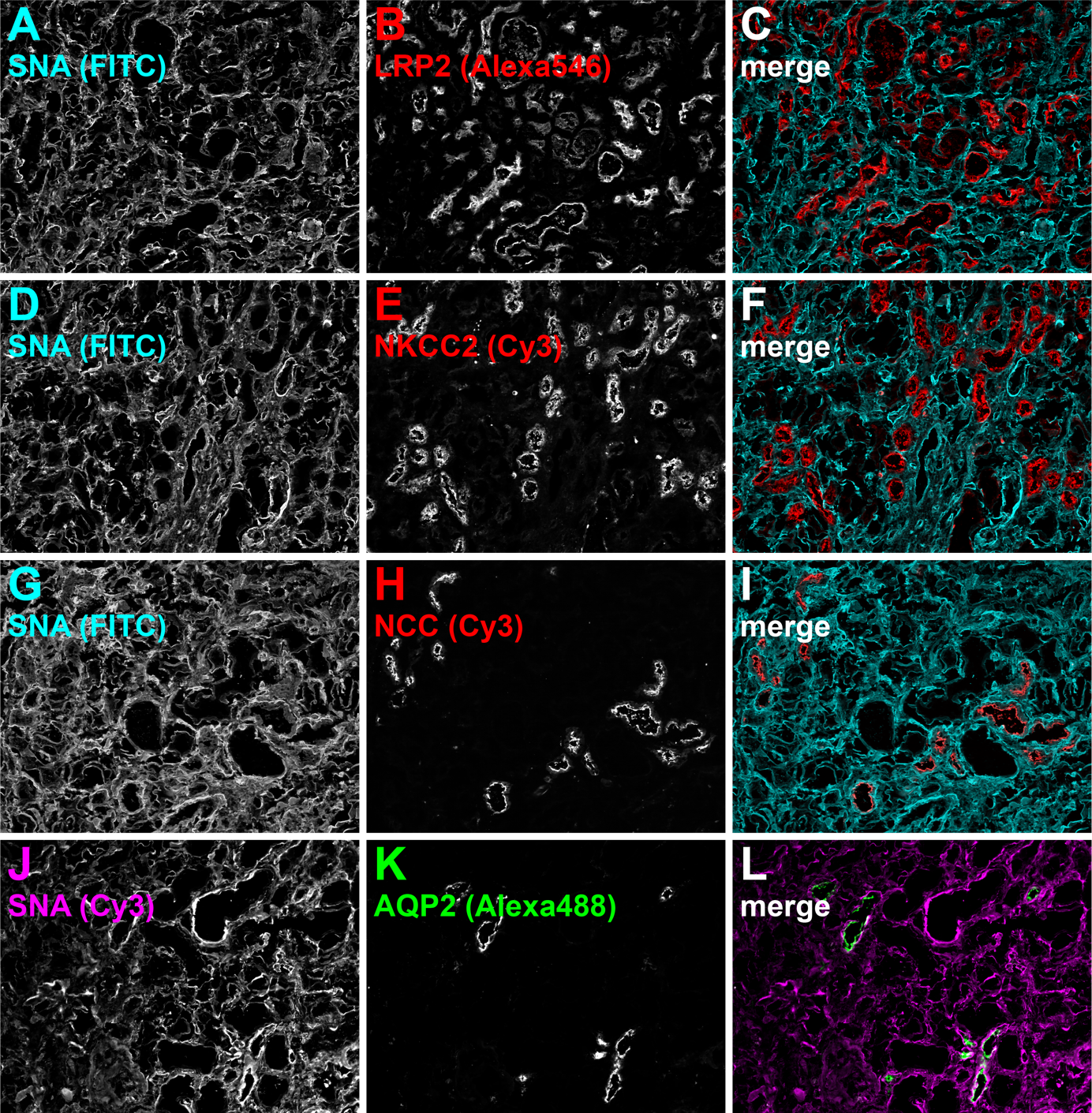


**Supplementary Fig. 12**. Single channels of SNA and nephron markers for human.

**Supplementary Tables**

Supplementary Table 1

| **Lectin/agglutinin** | **Fluorophore** | **Manufacturer** | **Catalog number** |
| --- | --- | --- | --- |
| Dolichos Biflorus Agglutinin (**DBA**) | Fluorescein | Invitrogen | L32474 |
| Dolichos Biflorus Agglutinin (**DBA**) | Rhodamine | Vectorlabs | RL-1032 |
| Jacalin | Fluorescein | Vectorlabs | FL-1151 |
| Lotus Tetragonolobus Lectin (**LTL**) | Fluorescein | Invitrogen | L32480 |
| Peanut Agglutinin (**PNA**) | Cy3 | Vectorlabs | CL-1073 |
| Soybean Agglutinin (**SBA**) | Alexa594 | Invitrogen | L32462 |
| Soybean Agglutinin (**SBA**) | Alexa647 | Invitrogen | L32463 |
| Soybean Agglutinin (**SBA**) | Fluorescein | Vectorlabs | FL-1011 |
| Soybean Agglutinin (**SBA**) | unconjugated | Vectorlabs | L1010 |
| Sambucus Nigra Lectin (**SNA**) | Cy3 | Vectorlabs | CL-1303 |
| Sambucus Nigra Lectin (**SNA**) | Fluorescein | Vectorlabs | FL-1301 |

Supplementary Table 2

| ***Gene*** | **Primer 1** | **Primer 2** |
| --- | --- | --- |
| *Gapdh* | AGCTTGTCATCAACGGGAAG | TTTGATGTTAGTGGGGTCTCG |
| *Nphs1* | CTGGGGGACAGTGGATTGAC | GTGTCTTCAGGAGCCTGGTC |
| *Nphs2* | TGGGGCATCAAAGTGGAGAG | CTTTGGCCTGTCTTTGTGCC |
| *Wt1* | CTGAAGACCCACACCAGGAC | GTCTGAGCGCGCAAACTTTT |
| *Lrp2* | GATGGATTAGCCGTGGACTG | TCCGTTGACTCTTAGCATCTGA |
| *Slc34a3* | CCTGCAGACATGTTAATCTTCG | AGACAGGCACCAGGTACCAC |
| *Slc12a1* | ATGCCTCGTATGCCAAATCT | CCCACATGTTGTAAATCCCATA |
| *Umod* | GAGACTGGATGTCCATAGTGACC | GCATGGGTTTCGTTTCTCC |
| *Slc12a3* | CCTCCATCACCAACTCACCT | CCGCCCACTTGCTGTAGTA |
| *Pvalb* | GGCAAGATTGGGGTTGAA | AGCAGTCAGCGCCACTTAG |
| *Trpm6* | CACAAGCCAGTGACCACCTA | TTCCATGTGGGGGTTTTATC |
| *Calb1* | GTGTGGGAAAGAGTTCAATAAGG | TTCTTCTCACACAGATCTTTCAGC |
| *Slc8a1* | CCATCCTAGGCGAGCACA | TCGTCTTCTTAATGAGTTTGTCCA |
| *Aqp2* | TAGCCCTGCTCTCTCCATTG | GAGCAGCCGGTGAAATAGAT |
| *Scnn1g* | TTATGTATAAGATGACTTGCAGACCA | AACAGAGAAAACGCCACCAT |
| *Slc26a4* | CTGGGGGACAGTGGATTGAC | GTGTCTTCAGGAGCCTGGTC |
| *GAPDH* | ACGGGAAGCTTGTCATCAAT | CATCGCCCCACTTGATTTT |
| *LRP2* | GCTTTATAGAGGGGAGCACCA | GGGCAGGTTGATAGGCAGT |
| *SLC34A1* | TCCAGAAGGTCATCAATACGG | GACCACGAAGGTCATGCTG |
| *SLC12A1* | GATGTGAACTGGGGCTCCTC | TGGGTCCCCCTGTTAAGACA |
| *UMOD* | CAGTCTCAAGGGCTTTTAGCA | TGAAAAGTCAGGGTCAAGGTG |
| *SLC12A3* | CTCTGCTGAGCAAGTTCCGA | TCAGACGGAAGGGTGCAATC |
| *PVALB* | CATCGAGGAGGATGAGCTGG | TTGTCTCCAGCAGCCATCAG |
| *TRPM6* | CCAGGAGCACCAACCTCAAT | GATGGTGAGGCTCTTGAGGG |
| *CALB1* | CCCTCATCACAGCCTCACAG | TGCAGCTCCTTTCCTTCCAG |
| *SLC8A1* | TCGTCGCACTTGGAACATCA | AGGAAGACATTCACCGCGTT |
| *AQP2* | ACCCCTGCTCTCTCCATAGG | GAGCAGCCGGTGTAATGGAT |
| *SCNN1G* | CCAATCAGGAACATCTACAACGC | TCTCCACCATCTTTGTCTGGAAG |
| *SLC26A4* | GGGGCTGGATCTCGGTTTAC | ATGCTTCCAAGGCCATTCCA |
